# Associations of three immune inflammatory markers with the risk of brain metastasis from lung cancer: a systematic review and meta-analysis

**DOI:** 10.3389/fonc.2026.1804811

**Published:** 2026-04-07

**Authors:** Mingfeng Liu, Yunze Shi, Anhao Chen, Xiaoyang Hu

**Affiliations:** 1 School of Graduate, Heilongjiang University of Chinese Medicine, Harbin, Heilongjiang, China; 2 The Second Clinical Medical College, Heilongjiang University of Chinese Medicine, Harbin, Heilongjiang, China; 3 Basic Medical College of Heilongjiang University of Chinese Medicine, Harbin, Heilongjiang, China

**Keywords:** brain metastasis, immune-inflammatory markers, lung cancer, meta-analysis, neutrophil-to-lymphocyte ratio, systematic review

## Abstract

**Objective:**

This study sought to comprehensively evaluate the associations between three immune-inflammatory markers, namely neutrophil-to-lymphocyte ratio (NLR), platelet-to-lymphocyte ratio (PLR), and lymphocyte-to-monocyte ratio (LMR), and the risk of brain metastasis from lung cancer (BMLC). Through a meta-analysis, this study was expected to offer evidence-based support for early clinical identification of high-risk patients.

**Methods:**

Databases including PubMed, Cochrane, EMBASE, Web of Science, and Scopus were comprehensively searched. Relevant articles published up to November 2025 were retrieved. Cohort studies investigating the associations between the above markers and BMLC were included. Two researchers independently screened the articles, extracted data, and assessed study quality. Stata 15.0 was utilized to perform the meta-analysis. A random-effects or fixed-effects model was applied to pool the effect sizes. Subgroup analyses, meta-regression, sensitivity analyses, and publication bias assessment were conducted.

**Results:**

Fourteen retrospective cohort studies were included, involving 3,643 participants with lung cancer. Pooled multivariate analyses revealed that elevated NLR served as an independent risk factor for BMLC (odds ratio [OR]=1.61, 95% confidence interval [CI]: 1.27–2.05, P_Z_<0.05). The association of NLR with BMLC was stronger in the small cell lung cancer (SCLC) subgroup (OR = 2.36, 95% CI: 1.60–3.47, P_Z_<0.05) and the metachronous brain metastasis (MBM) subgroup (OR = 1.84, 95% CI: 1.19–2.85, P_Z_<0.05). Pooled univariate analyses revealed that low LMR was associated with an elevated risk of brain metastasis (BM) (OR = 1.24, 95% CI: 1.03–1.49, P<0.05). Multivariate analyses incorporating original studies further suggested that the association between LMR and the risk of BM was more significant only in the non-small cell lung cancer (NSCLC) subgroup and synchronous brain metastasis (SBM) subgroup. PLR showed no significant association with the risk of BM in the overall analysis.

**Conclusion:**

Existing evidence indicates that high NLR before treatment is an independent risk factor for BMLC, with a stronger association in patients with SCLC. The association between low LMR and the risk of BM is context-specific, primarily observed in patients with NSCLC and SBM. These findings provide evidence-based support for stratifying the risk of BM with routine inflammatory markers, warranting further validation in prospective studies.

**Systematic Review Registration:**

https://www.crd.york.ac.uk/PROSPERO/myprospero, identifier CRD420251170550.

## 1 Introduction

Lung cancer (LC) is one of the most common and fatal malignancies worldwide (1). Brain metastasis (BM) represents a prevalent type of distant metastasis of LC (2). Once BM occurs, the prognosis can be extremely poor, with a significantly shortened median overall survival, typically less than one year. BM from LC (BMLC) is accompanied by symptoms such as headaches, cognitive impairment, and neurological deficits, which seriously impact patients’ quality of life, significantly elevating treatment difficulty and disease burden (3, 4). Therefore, identifying patients at high risk for BM early is critical for developing individualized treatment strategies, optimizing follow-up plans, and improving prognosis. However, effective early-stage biomarkers for BM remain lacking in clinical practice. BM is primarily diagnosed by imaging (5), which shows findings lagging behind pathological changes and is incapable of dynamic monitoring.

Recent research has uncovered that systemic inflammatory responses are closely linked to the formation, progression, and metastasis of tumors (6, 7). The underlying mechanism is that tumor-associated inflammation induces proliferation and activation of immune cells (e.g., neutrophils, monocytes) while causing functional exhaustion of lymphocytes (8). This imbalance not only accelerates the progression of primary tumors but also disrupts the blood-brain barrier and suppresses central immune surveillance through inflammatory mediators, thereby creating a microenvironment conducive to the colonization of lung cancer cells in the brain (9, 10). Hence, it is clinically imperative to identify biomarkers capable of identifying high-risk patients before symptoms appear. As part of routine blood tests, white blood cell differential count yields multiple inflammatory indices, including the neutrophil-to-lymphocyte ratio (NLR), platelet-to-lymphocyte ratio (PLR), and lymphocyte-to-monocyte ratio (LMR). These indices reflect the dynamic equilibrium between antitumor immunity and the proinflammatory microenvironment (11). Their associations with the risk of tumor progression and metastasis have been detected in multiple types of cancer (12). However, a systematic assessment of the association between these inflammatory indices and BMLC remains lacking.

Given the varying quality of evidence, a systematic review may inform evidence-based decision-making. Through a systematic search, rigorous evaluation, and quantitative synthesis, existing studies are efficiently integrated to enhance the statistical power and identify potential sources of heterogeneity, thereby yielding more robust and definitive conclusions. Therefore, this review adopted a meta-analysis approach to synthesize existing evidence, aiming to examine the associations of NLR, PLR, and LMR with BMLC and offer evidence-based guidance for clinical risk stratification.

## 2 Materials and methods

### 2.1 Literature search

This review was registered in the International Prospective Register of Systematic Reviews (PROSPERO) with ID CRD420251170550. It was conducted following the Preferred Reporting Items for Systematic Reviews and Meta-Analyses (PRISMA) 2020 statement (13).

Two researchers independently searched five databases, namely PubMed, Cochrane, EMBASE, Web of Science, and Scopus, for studies examining the impact of NLR, PLR, and LMR on BMLC. The literature search covered articles published up to November 2025. Search strings comprised the subject headings “lung cancer,” “brain,” “metastasis,” “NLR,” “PLR,” and “LMR,” along with a broad range of free-text terms. Detailed database-specific search strategies are provided in the **Supplementary Table 1**: Search Strategy.

All studies were examined by the researchers independently for possible eligibility, and any discrepancies were settled by discussion until an agreement was achieved.

### 2.2 Inclusion and exclusion criteria

Inclusion criteria comprised: (i) Population: Studies enrolling patients diagnosed with lung cancer (regardless of age, sex, race, or clinical staging); (ii) Definition of indices: Studies reporting lymphocyte-related indices at baseline. Baseline was explicitly defined in this study as blood samples collected at admission or before treatment. The indices included neutrophil-to-lymphocyte ratio (NLR), platelet-to-lymphocyte ratio (PLR), and lymphocyte-to-monocyte ratio (LMR). Studies categorizing patients into high- and low-level groups by self-defined cutoff values were included. (iii) Outcome: The primary outcome was BM, which must be confirmed by imaging (magnetic resonance imaging [MRI] or computerized tomography). Based on the temporal relationship between BM and the diagnosis of LC, and referencing previous studies, synchronous brain metastasis (SBM) was defined as BM detected at the first diagnosis of LC, while metachronous brain metastasis (MBM) was defined as BM newly occurring during the disease course after the definitive diagnosis of LC (14). Studies analyzing the relationship between these indices and BMLC and directly or indirectly providing corresponding effect sizes and their 95% confidence intervals (CI) were included; (iv) Study design: Given that this study was intended to explore whether NLR, PLR, and LMR were independent risk factors for BMLC, only cohort studies (CSs) or randomized controlled trials (RCTs) may be included.

Exclusion criteria comprised: (i) Non-human studies (e.g., animal studies), technical reports, letters to the editor, case reports, conference abstracts, books, clinical guidelines, meta-analyses, or reviews; (ii) Articles without available full texts; (iii) Studies with no extractable data or with non-relevant data.

### 2.3 Literature screening and quality assessment

Two researchers screened the retrieved records independently following the predefined eligibility criteria. Initial search records were loaded into EndNote 20, which served as the literature management tool for this study. Two researchers independently identified duplicate records, screened the titles and abstracts, and further evaluated the studies for eligibility by downloading and reviewing full texts.

Two researchers assessed the quality of the articles independently through full-text review. Ultimately, only cohort studies were included, which were scored with the Newcastle-Ottawa Scale (NOS), a scale applicable for case-control or cohort studies (15). The scale primarily assessed three aspects: selection of study population, between-group comparability, and measurement of outcomes. High quality was defined by a score of 7 or more stars, moderate quality by 4–6 stars, and low quality by fewer than 4 stars.

Any ambiguities arising from the above research process and its results were resolved through discussion among all researchers.

### 2.4 Data extraction

Utilizing a pre-specified standardized data extraction form, two researchers independently extracted the following information from each study: (i) Basic characteristics of original studies: first author, publication year, age, country, study design; (ii) Subject characteristics: LC subtype, total sample size, number of patients with BM, biomarker, time of biomarker measurement, and type of BM. Additionally, to conduct a comprehensive analysis and ensure the robustness of the results, we extracted effect size data from both univariate (unadjusted) and multivariate (adjusted) analyses from the included studies. Univariate analyses facilitated initial identification of associations, while multivariate analyses enabled more accurate estimation of the independent associations between exposure and outcomes through control for potential confounders (e.g., country, age, LC subtype), thereby enhancing internal validity. For studies reporting hazard ratios (HRs), we adopted the method by Shor et al. to estimate the corresponding risk ratios (RRs) (16). Considering that all original studies were CSs, the estimated RR values were considered to be approximately equivalent to the corresponding odds ratio (OR) values (17). Effect sizes and the lower and upper limits of confidence intervals were log-transformed to facilitate subsequent meta-analysis. Any ambiguities arising from the process or results of data extraction were addressed by discussion among all researchers. If data were presented only graphically, Engauge Digitizer (version 12.1) was utilized for digitization and extraction of numerical values.

Any disagreements arising in the above steps were settled by discussion with a third researcher.

### 2.5 Statistical analysis

Data synthesis and statistical analysis were performed utilizing StataSE 15.0. The ORs and 95% confidence intervals (95% CIs) served as effect sizes to measure the associations between high NLR and PLR and low LMR with BMLC. Heterogeneity among studies was assessed utilizing Cochran’s Q test and Higgins’ I^2^ statistic (18). When I^2^ > 50% and P_H_ < 0.1, significant heterogeneity existed, and a random-effects model was therefore applied. Otherwise, a fixed-effects model was adopted. When the number of studies was insufficient, a random-effects model was still utilized to prevent overconfidence in the findings. Forest plots were generated to display the effect sizes and their confidence intervals across studies. To explore potential sources of any heterogeneity in the pooled results, subgroup analyses were performed on data grouped by LC subtype (non-small cell lung cancer [NSCLC] vs. small cell lung cancer [SCLC]), geographical region (Asian vs. Non-Asian), and type of BM (SBM vs. MBM) to assess the associations of high NLR and PLR and low LMR with BMLC. Furthermore, meta-regression analysis was employed to investigate whether study characteristics contributed to heterogeneity. Additionally, sensitivity analyses were also implemented by sequentially excluding studies to evaluate the stability of the pooled results. The Egger’s test was employed to detect publication bias (19). Finally, the trim-and-fill method was adopted to adjust for potential publication bias (20) to ensure reliable and accurate results. This study included subgroup analyses based on data sources. If data were insufficient, analyses were not performed, or appropriate adjustments were made. Publication bias and funnel plots were analyzed when supported by the available data.

## 3 Results

### 3.1 Literature screening results

**Figure 1:**
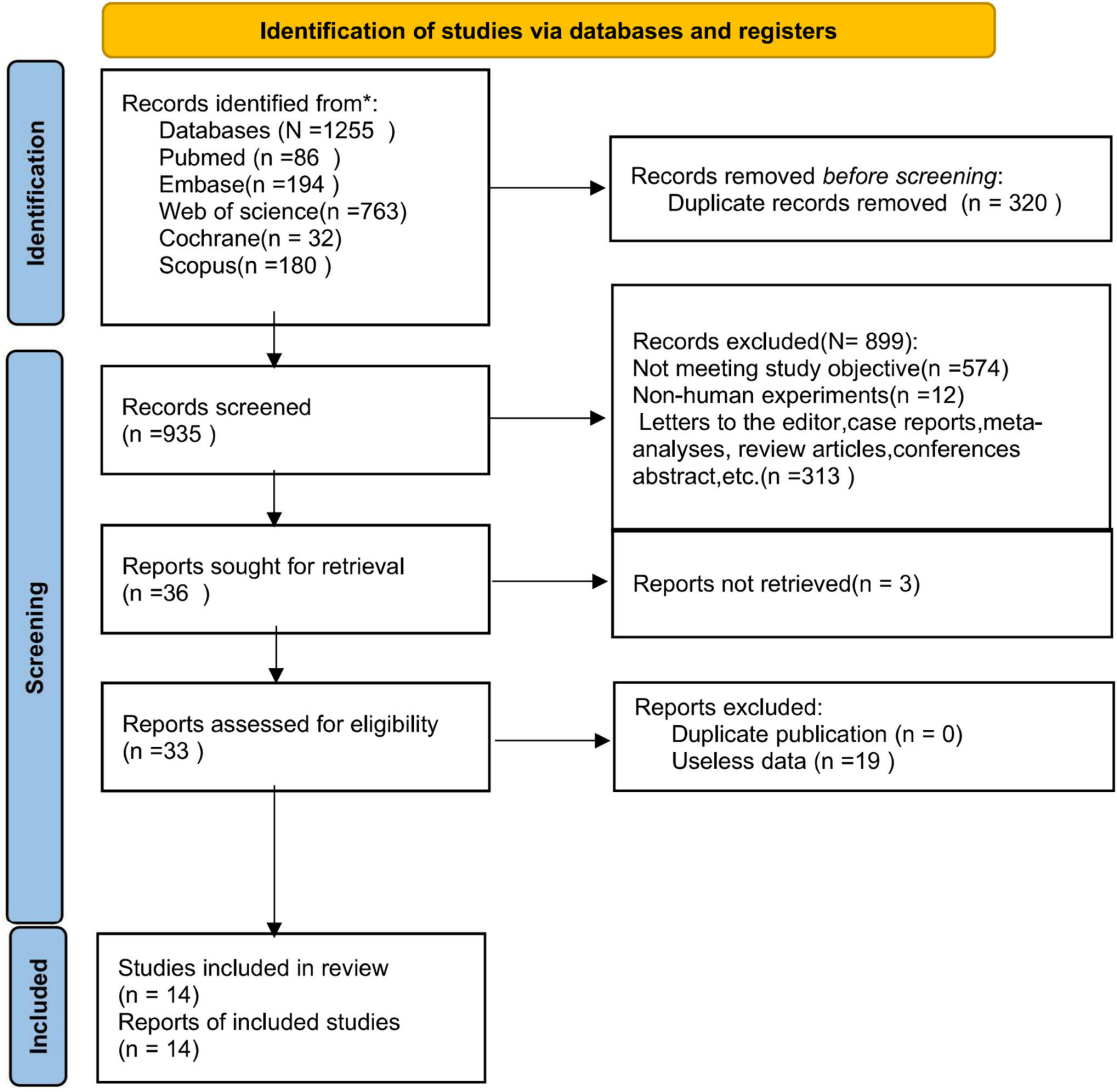
Literature screening flowchart (PRISMA flowchart).

Initial searches on the five databases yielded 1,255 studies. Of these, 320 were excluded due to duplication. Subsequently, 902 studies were removed according to the predefined eligibility criteria, and 19 studies were eliminated during full-text assessment. Detailed reasons for exclusion and the screening process are depicted in **Figure 1**.

### 3.2 Basic characteristics of included studies

This meta-analysis ultimately included fourteen retrospective cohort studies (RCSs) (21–34), which involved a total of 3,643 patients with LC and reported 1,152 BM events.

Among these studies, twelve studies originated from Asia (21, 23–33), one from Europe (22), and one from North America (34). Seven studies enrolled patients with SCLC (21–23, 29, 31, 33, 34), while eight studies enrolled patients with NSCLC (22, 24–28, 30, 32). Six studies examined SBM (23–25, 27, 30, 32), while nine studies examined MBM (21, 22, 26, 28–31, 33, 34). Ten studies investigated the relationship between NLR and BMLC, eight studies investigated the relationship between PLR and BMLC, and six studies examined the relationship between LMR and BMLC. Detailed characteristics of each study are listed in **Table 1**.

**Table 1:** Detailed characteristics of included studies.

Author	Year	Country	Study design	Cancer subtype	Sample size	Age (mean/median)	Patients with BM	Biomarker	Time of biomarker measurement	Type of BM
Yuntao Zhou	2025	China	RCS	SCLC	141	59.03 ± 9.32	46	LMR	baseline	MBM
Karol Marschollek	2025	Poland	RCS	SCLC, NSCLC	128	41-91	41	NLR, PLR, LMR	baseline	MBM
Weiwei Li	2023	China	RCS	SCLC	631	60.30 ± 8.92	103	NLR, PLR, LMR	baseline	SBM
I Abuelbeh	2022	Jordan	RCS	NSCLC	722	Median age 59	280	NLR	baseline	SBM
Yafeng Liu	2022	China	RCS	NSCLC	228	N/A	63	LMR	baseline	SBM
Wei Wang	2017	China	RCS	NSCLC	103	60.61 ± 8.57	12	PLR	baseline	MBM
Young Wha Koh	2016	South Korea	RCS	NSCLC	260	59.35 ± 9.57	60	NLR	baseline	SBM
F Sert	2021	Turkey	RCS	NSCLC	208	62.66 ± 9.83	56	NLR, PLR	baseline	MBM
Joo-Hyun Chung	2020	South Korea	RCS	SCLC	137	69.59 ± 9.01	28	NLR, PLR	baseline	MBM
Weiguo Gu	2024	China	RCS	NSCLC	236	N/A	122	NLR, PLR	baseline	SBM, MBM
Qing Hou	2022	China	RCS	SCLC	246	N/A	104	LMR	baseline	MBM
Chunxiao Hu	2022	China	RCS	NSCLC	210	N/A	56	NLR, PLR, LMR	baseline	SBM
Jianjian Qiu	2022	China	RCS	SCLC	100	N/A	32	NLR, PLR	baseline	MBM
Ryoko Suzuki	2018	USA	RCS	SCLC	293	64.03 ± 2.27	115	NLR, PLR	baseline	MBM

RCS: retrospective cohort study; N/A: Not available; SCLC: small cell lung cancer; NSCLC: non-small cell lung cancer; NLR: neutrophil-to-lymphocyte ratio; PLR: platelet-to-lymphocyte ratio; LMR: lymphocyte-to-monocyte ratio; SBM: synchronous brain metastasis; MBM: metachronous brain metastasis.

### 3.3 Quality assessment results

Quality assessment of the included RCSs utilizing the NOS suggested overall high methodological quality. Among the fourteen RCSs, twelve studies were rated as high quality, including seven studies assigned with 8 stars and five studies assigned with 7 stars. The other two studies were assigned 6 stars, primarily due to enrollment of patients with pre-existing BM (SBM) at baseline. However, considering that BM is common in the early stage of LC, the actual methodological rigor of these studies may exceed the level reflected by the scale scores (**Supplementary Table 2**).

### 3.4 Meta-analysis results

#### 3.4.1 Association between NLR and BMLC

Eight articles (22–24, 27, 29, 30, 32, 34) provided results of univariate analyses on the association between NLR and BMLC. The heterogeneity test yielded I^2^ = 59.9% and P_H_ < 0.05, indicating significant heterogeneity. Therefore, data were pooled with a random-effects model. Meta-analysis suggested that compared to low NLR, high NLR was significantly linked to BMLC (OR = 1.13, 95% CI: 1.03-1.23, Z = 2.73, P_Z_ < 0.05). The forest plot is displayed in **Supplementary Figure 1**. Publication bias assessment (P = 0.086) demonstrated robustness of this finding, as shown in **Supplementary Figure 2**. The funnel plot is presented in **Supplementary Figure 3**.

**Figure 2:**
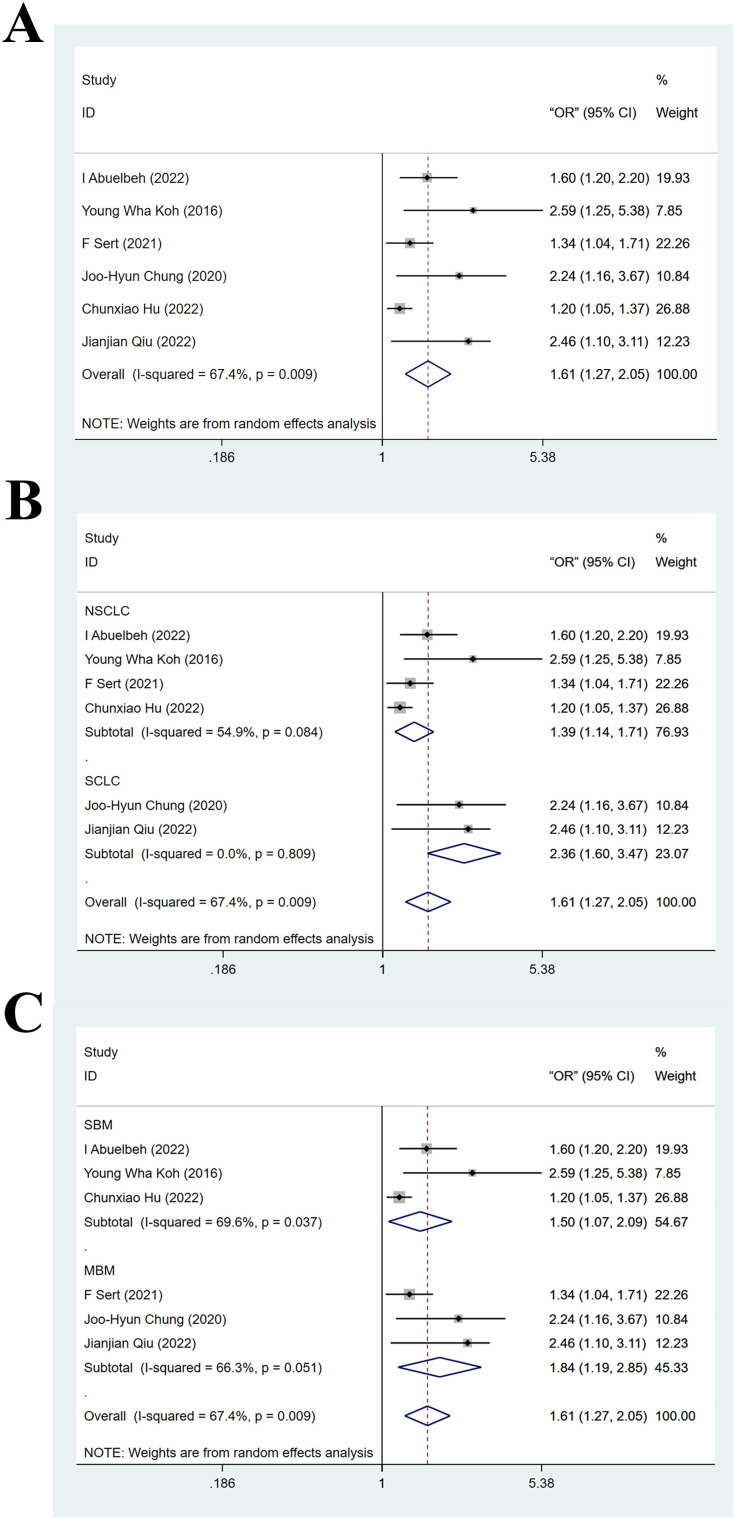
**(A)** Forest plot of multivariate analyses on the association between high neutrophil-to-lymphocyte ratio and brain metastasis from lung cancer; **(B)** Forest plot of subgroup analysis on the association between high neutrophil-to-lymphocyte ratio and brain metastasis from lung cancer (stratified by subtype of lung cancer); **(C)** Forest plot of subgroup analysis on the association between high neutrophil-to-lymphocyte ratio and brain metastasis from lung cancer (stratified by type of brain metastasis).

Six articles (24, 27–29, 32, 33) provided results of multivariate analyses on the association between NLR and BMLC. The heterogeneity test yielded I^2^ = 67.4% and P_H_ < 0.05, indicating significant heterogeneity. Therefore, data were pooled with a random-effects model. Results suggested that high NLR was markedly linked to an elevated risk of BMLC (OR = 1.61, 95% CI: 1.27-2.05, Z = 3.93, P_Z_ < 0.05). The forest plot is displayed in **Figure 2A**. Sensitivity analysis demonstrated robustness of this finding, as shown in **Supplementary Figure 4**.

Subgroup analyses based on LC subtype and BM type were conducted on the above pooled results from multivariate analyses. In the NSCLC subgroup, the pooled effect size for the risk of BM in the high NLR cohort was OR = 1.39 (95% CI: 1.14–1.71, Z = 3.20, P_Z_ < 0.05). In the SCLC subgroup, this effect size increased to OR = 2.36 (95% CI: 1.60–3.47, Z = 4.35, P_Z_ < 0.05). This suggested that high NLR was linked to a higher risk of BM in SCLC patients compared to NSCLC patients, as shown in **Figure 2B**. In the SBM subgroup, the pooled effect size for the risk of BM in the high NLR cohort was OR = 1.50 (95% CI: 1.07–2.09, Z = 2.36, P_Z_ < 0.05). In the MBM subgroup, this effect size was OR = 1.84 (95% CI: 1.19–2.85, Z = 2.72, P_Z_ < 0.05). This suggested that high NLR is associated with BMLC regardless of the disease stage, as shown in **Figure 2C**. Meta-regression analysis demonstrated that pathological subtype of LC (P = 0.231), country (P = 0.488), and BM type (P = 0.485) were not sources of significant heterogeneity.

#### 3.4.2 Association between PLR and BMLC

Seven articles (22, 23, 26, 29, 30, 32, 34) provided results of univariate analyses on the association between PLR and BMLC. The heterogeneity test yielded I^2^ = 72.9% and P_H_ < 0.05, indicating significant heterogeneity. Therefore, data were pooled with a random-effects model. Meta-analysis suggested that compared to low PLR, high PLR was not significantly associated with BMLC (OR = 1.00, 95% CI: 1.00-1.01, Z = 0.58, Pz > 0.05). The forest plot is displayed in **Supplementary Figure 5**. Publication bias assessment (P = 0.081) demonstrated robustness of this finding, as shown in **Supplementary Figure 6**. The funnel plot is presented in **Supplementary Figure 7**.

**Figure 3:**
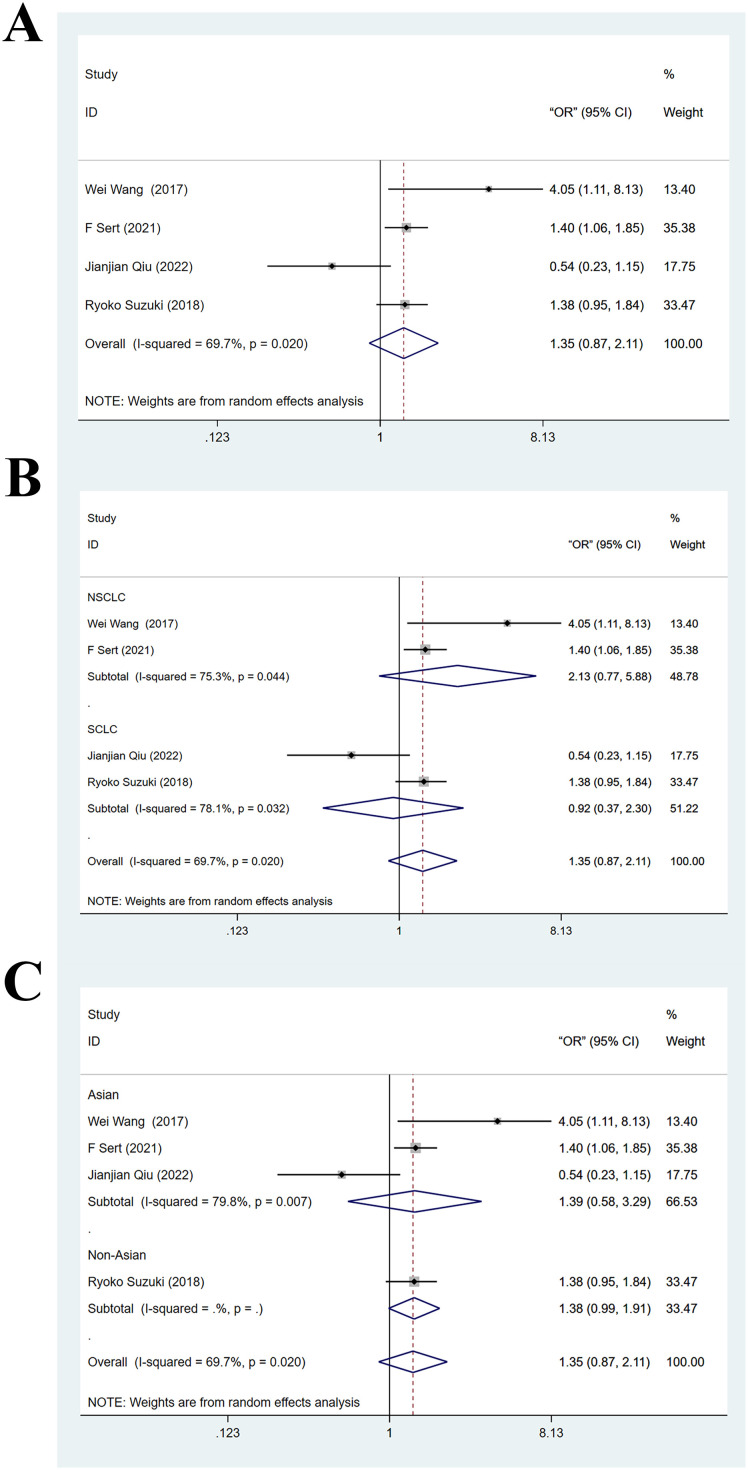
**(A)** Forest plot of multivariate analyses on the association between high platelet-to-lymphocyte ratio and brain metastasis from lung cancer; **(B)** Forest plot of subgroup analysis on the association between high platelet-to-lymphocyte ratio and brain metastasis from lung cancer (stratified by subgroup of lung cancer); **(C)** Forest plot of subgroup analysis on the association between high platelet-to-lymphocyte ratio and brain metastasis from lung cancer (stratified by country).

Four articles (26, 28, 33, 34) provided the results of multivariate analyses on the association between PLR and BMLC. The heterogeneity test yielded I^2^ = 69.7% and PH < 0.05, indicating significant heterogeneity. Therefore, data were pooled with a random-effects model. Results showed no significant association between high PLR and BMLC (OR = 1.35, 95% CI: 0.87-2.11, z = 1.34, Pz > 0.05). The forest plot is displayed in **Figure 3A**. Sensitivity analysis demonstrated robustness of this finding (**Supplementary Figure 8**).

Subgroup analyses based on LC subtype and country were conducted on the above pooled results from multivariate analyses. In the NSCLC subgroup, the pooled effect size for the risk of BM in the high PLR cohort was OR = 2.13 (95% CI: 0.77–5.88, Z = 1.46, Pz > 0.05). In the SCLC subgroup, this effect size was OR = 0.92 (95% CI: 0.37–2.30, Z = 0.17, Pz > 0.05). This suggested that high PLR was not significantly linked to BMLC, as shown in **Figure 3B**. In the Asian studies, the pooled effect size for the risk of BM in the high PLR cohort was OR = 1.39 (95% CI: 0.58–3.29, Z = 0.74, Pz > 0.05). In the non-Asian studies, this effect size was OR = 1.38 (95% CI: 0.99–1.91, Z = 1.89, Pz > 0.05). This suggested that high PLR was not significantly linked to BMLC, as shown in **Figure 3C**. Meta-regression analysis revealed that neither the pathological subtype of LC (P = 0.377) nor country (P = 0.526) was a source of significant heterogeneity.

#### 3.4.3 Association between LMR and BMLC

Six articles (21–23, 25, 31, 32) provided results of univariate analyses on the association between LMR and BMLC. The heterogeneity test yielded I^2^ = 63.9% and PH < 0.05, indicating significant heterogeneity. Therefore, data were pooled with a random-effects model. Meta-analysis suggested that, compared to high LMR, low LMR was linked to an elevated risk of BMLC (OR = 1.24, 95% CI: 1.03-1.49, Z = 2.30, P_Z_ < 0.05). The forest plot is displayed in **Supplementary Figure 9**.

**Figure 4:**
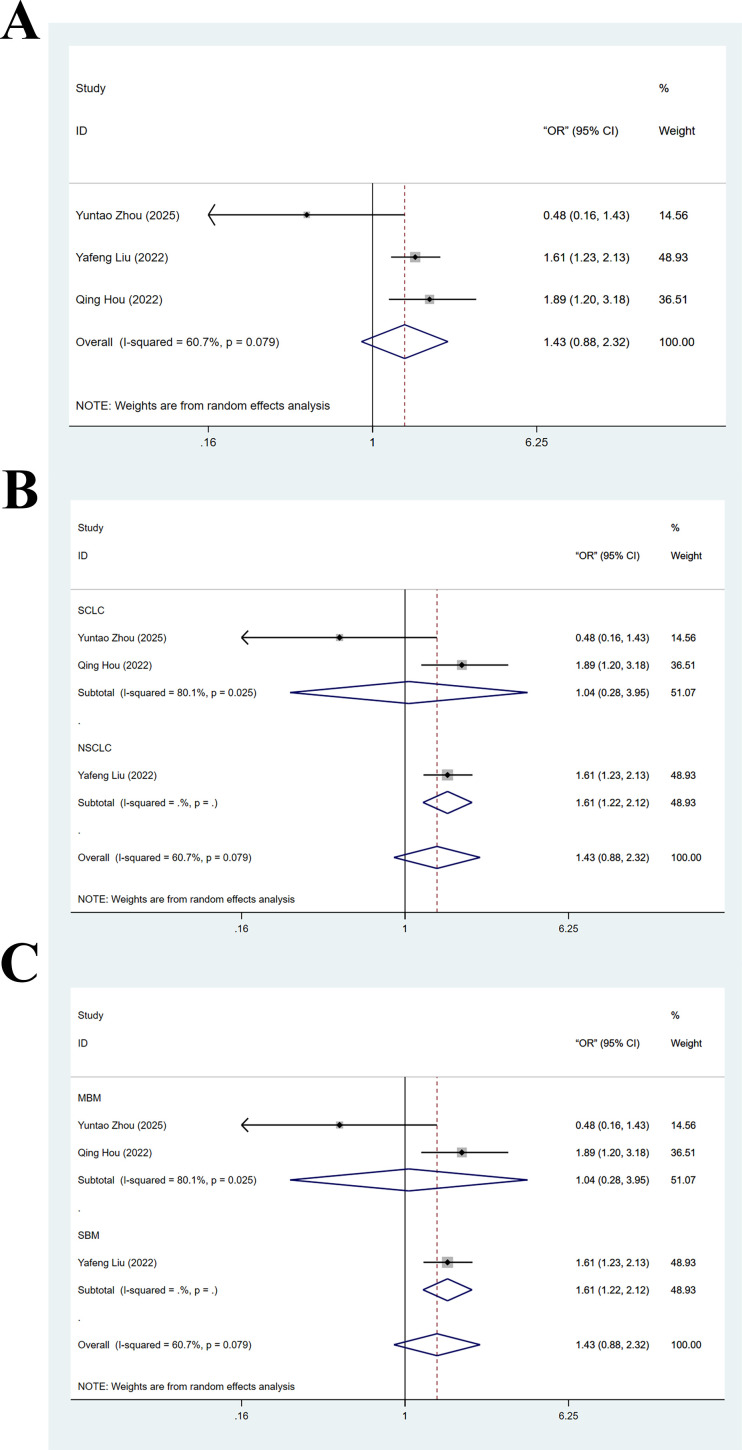
**(A)** Forest plot of multivariate analyses on the association between low lymphocyte-to-monocyte ratio and brain metastasis from lung cancer; **(B)** Forest plot of subgroup analysis on the association between low lymphocyte-to-monocyte ratio and brain metastasis from lung cancer (stratified by subtype of lung cancer); **(C)** Forest plot of subgroup analysis on the association between low lymphocyte-to-monocyte ratio and brain metastasis from lung cancer (stratified by type of brain metastasis).

Three articles (21, 25, 31) provided results of multivariate analyses on the association between LMR and BMLC. The heterogeneity test yielded I^2^ = 60.7% and P_H_ > 0.05, indicating significant heterogeneity. Therefore, data were pooled with a random-effects model. Results suggested that low LMR was not significantly linked to BMLC (OR = 1.43, 95% CI: 0.88-2.32, Z = 1.46, Pz > 0.05). The forest plot is displayed in **Figure 4A**. Sensitivity analysis demonstrated robustness of this finding (**Supplementary Figure 10**).

Subgroup analyses based on LC subtype and BM type were conducted on the above pooled results from multivariate analyses. In the NSCLC subgroup, the pooled effect size for the risk of BM in the low LMR cohort was OR = 1.61 (95% CI: 1.22–2.12, Z = 3.40, P_Z_ < 0.05). In the SCLC subgroup, this effect size was OR = 1.04 (95% CI: 0.28–3.95, Z = 0.06, Pz > 0.05). This suggested that compared to high LMR, low LMR was linked to an elevated risk of BM in patients with NSCLC, but not in patients with SCLC (**Figure 4B**). In the SBM subgroup, the effect size for the risk of BM in the low LMR cohort was OR = 1.61 (95% CI: 1.22–2.12, Z = 3.40, P_Z_ < 0.05). In the MBM subgroup, this effect size was OR = 1.04 (95% CI: 0.28–3.95, Z = 0.06, P_Z_ > 0.05). This suggested that compared to high LMR, low LMR was linked to an elevated risk of BM in early-stage LC (**Figure 4C**).

## 4 Discussion

This systematic review investigated the association between systemic inflammatory indices derived from lymphocyte-related markers and BMLC through a meta-analysis, aiming to provide evidence-based support for addressing the symptoms, treatment challenges, and disease burden that severely impact the quality of life following BM. By pooling results from univariate and multivariate analyses, it was revealed that only NLR served as an independent risk factor for BMLC after accounting for key confounders. In contrast, evidence for independent associations of PLR and LMR with BM was limited in the overall population. These findings are further discussed below.

The pooled results from univariate analyses demonstrated the association of high NLR with an elevated risk of BM (OR = 1.13). This association was strengthened after pooling results from multivariate analyses (OR = 1.61), indicating that the significance of NLR as a risk factor was not influenced by confounders. This finding aligns with the hypothesis that systemic inflammation is a common driver of tumor metastasis (35). The underlying biological mechanism lies in the fact that when NLR increases, the activity of pro-metastatic neutrophils is enhanced, which disrupts the vascular barrier and facilitates tumor cell colonization through mechanisms such as the release of proteases and the formation of extracellular traps. Meanwhile, the function of anti-tumor lymphocytes is diminished (36, 37). These dual pathological processes create a systemic environment conducive to brain metastasis. Future translational research is needed to elucidate the specific molecular and immune pathways through which NLR promotes brain metastasis and to explore its potential as a therapeutic target. Subgroup analyses further revealed a stronger association of NLR with the risk of BM in SCLC patients (OR = 2.36) than in NSCLC patients (OR = 1.39). This finding aligns with the inherent higher aggressiveness and propensity for brain metastasis of SCLC (38), suggesting that systemic inflammatory responses may contribute more to the rapid progression of SCLC. Therefore, for patients with SCLC, baseline NLR levels may serve as a consideration for risk stratification. The strong association between NLR and the risk of brain metastasis suggests that implementing more frequent cranial imaging follow-up for patients with elevated NLR levels could be a clinical strategy worth exploring. However, the benefits of this strategy still need to be further validated by prospective studies. Furthermore, high NLR was associated with an elevated risk of both SBM and MBM (SBM: OR = 1.50; MBM: OR = 1.84), with a potentially higher effect size for MBM. This may stem from the role of NLR as a dynamic indicator that continuously reflects tumor-induced systemic inflammation (39). As the disease progresses, this chronic inflammation persistently drives metastasis. Consequently, the association of NLR with BM occurring in the advanced stage becomes stronger. This suggests that monitoring the dynamic changes in NLR may be conducive to the continuous assessment of the risk of metastasis in the long term, which is superior to baseline evaluation alone. Based on the above discussion, routine NLR test before treatment serves as a low-cost, readily accessible risk assessment tool that facilitates the identification of patients at high risk for BM. For patients with elevated NLR levels, clinicians may consider intensifying follow-up monitoring with cranial MRI or incorporating the high risk of brain metastasis into the comprehensive consideration when developing treatment plans.LMR exhibited a distinct association with BMLC. Pooled results from univariate analyses revealed that low LMR (rather than high LMR) was linked to a heightened risk of BMLC (OR = 1.24). However, evidence supporting it as an independent risk factor was insufficient in pooled multivariate analyses. Nevertheless, subgroup analyses revealed its significant clinical specificity. Low LMR demonstrated significant predictive value for the risk of BM in NSCLC and SBM subgroups. This suggests that a lower LMR at baseline, reflecting a relative reduction in lymphocytes, may be associated with diminished systemic anti-tumor immune responses, which impairs the body’s ability to eliminate or suppress early micrometastases in NSCLC (40). Therefore, pre-treatment assessment of LMR may be conducive to identifying a high risk of BM in NSCLC patients. Clinically, LMR can be useful for identifying high risk of BM in early-stage NSCLC patients, thereby guiding intensified monitoring during the initial treatment phase.

This study revealed no significant independent association between PLR and BMLC. This association was statistically insignificant in both multivariate analyses and subgroup analyses. This insignificance may stem from the relatively low specificity of platelet count, which is susceptible to interference from multiple non-tumor-related factors such as inflammation, medications, and coagulation (41). Although the predictive role of PLR for the risk of BMLC was not significant, the trend was consistent, and its value may become apparent in larger-scale studies. Therefore, existing evidence disproves the usability of PLR alone for assessing the risk of BM.

## 5 Limitations

First, all the studies included are RCSs due to the lack of prospective studies. However, all studies adopt a cohort design, which to some extent ensures that inflammatory levels are measured prior to BM, thereby establishing a relatively clear temporal relationship. Additionally, quality assessment is conducted rigorously utilizing the NOS scale, and sensitivity analyses are performed to confirm the robustness of primary findings. These procedures enhance the reliability of the evidence offered by this study. To validate the findings presented in this systematic review, future prospective cohort studies should be conducted, which should employ standardized collection of baseline inflammatory markers and long-term follow-ups to accurately assess the predictive efficacy of these markers for MBM and establish reliable clinical thresholds. Besides, methodological limitations in evidence synthesis exist. Due to the limited number of studies included, statistical methods are insufficient to effectively assess publication bias for some inflammatory markers. Future high-quality studies are warranted to enhance the robustness of the findings. Additionally, the standardization of effect sizes in this meta-analysis involved converting HRs to risk ratios RRs, and subsequently approximating RRs as ORs. It is crucial to recognize that HRs, RRs, and ORs represent distinct measurement metrics. Such conversions may introduce bias and potentially compromise the accuracy of pooled estimates. Therefore, findings based on effect sizes derived from these conversions should be interpreted with caution. Finally, significant heterogeneity is observed in some pooled analyses. Nonetheless, we have assessed and addressed such heterogeneity by employing a random-effects model, subgroup analyses, and sensitivity analyses, which have enhanced the statistical robustness of the findings.

## 6 Conclusion

This meta-analysis systematically evaluated the association between three immune-inflammatory markers and BMLC. The results revealed that high NLR before treatment was significantly associated with an elevated risk of BMLC. This association was particularly evident in SCLC and MBM subgroups, suggesting the potential value of NLR for risk stratification. Although LMR showed no significant association with BMLC in the overall population, subgroup analyses suggested LMR may be useful for identifying high risk of BM in early-stage NSCLC patients. In contrast, PLR exhibited no consistent association with BMLC. These exploratory findings warrant attention and validation in future research.

## Data Availability

The original contributions presented in the study are included in the article/supplementary material. Further inquiries can be directed to the corresponding author.
