# Supplementary material for: Associations of three immune inflammatory markers with the risk of brain metastasis from lung cancer: a systematic review and meta-analysis
Source: Front Oncol. 2026 Apr 7;16:1804811. doi: 10.3389/fonc.2026.1804811 (PMC13096944; doi:10.3389/fonc.2026.1804811)
Supplement: Supplementary file 1 [file DataSheet1.docx]

**Table S1.** Search strategy

| **Pubmed** | |
| --- | --- |
| # | Query |
| 1 | "Neoplasm Metastasis"[MeSH Terms] |
| 2 | "cancer cell dissemination"[Title/Abstract] OR "cancer cell spread"[Title/Abstract] OR "cancer dissemination"[Title/Abstract] OR "cancer spread"[Title/Abstract] OR "disseminated tumor cell"[Title/Abstract] OR "disseminated tumour cell"[Title/Abstract] OR "Metasta*"[Title/Abstract] OR "neoplastic cell dissemination"[Title/Abstract] OR "secondary cancer"[Title/Abstract] OR "secondary carcinoma"[Title/Abstract] OR "tumor dissemination"[Title/Abstract] OR "tumor migration"[Title/Abstract] OR "tumor spread"[Title/Abstract] OR "tumour dissemination"[Title/Abstract] OR "tumour migration"[Title/Abstract] OR "tumour spread"[Title/Abstract] |
| 3 | ("Brain"[MeSH Terms]) OR ("Cerebrum"[MeSH Terms]) |
| 4 | "Brain*"[Title/Abstract] OR "Cerebr*"[Title/Abstract] OR "encephalon"[Title/Abstract] |
| 5 | (("Lung Neoplasms"[MeSH Terms]) OR ("Small Cell Lung Carcinoma"[MeSH Terms])) OR ("Carcinoma, Non-Small-Cell Lung"[MeSH Terms]) |
| 6 | "bronchial non small cell cancer"[Title/Abstract] OR "bronchial non small cell carcinoma"[Title/Abstract] OR "bronchial small cell cancer"[Title/Abstract] OR "bronchial small cell carcinoma"[Title/Abstract] OR "bronchopulmonary cancer"[Title/Abstract] OR "broncho-pulmonary cancer"[Title/Abstract] OR "bronchopulmonary neoplas*"[Title/Abstract] OR "broncho-pulmonary neoplasm"[Title/Abstract] OR "bronchopulmonary tumor"[Title/Abstract] OR "broncho-pulmonary tumor"[Title/Abstract] OR "Ca lung"[Title/Abstract] OR "Cancer of Lung"[Title/Abstract] OR "cancer of the lung"[Title/Abstract] OR "carcinogenesis of the lung"[Title/Abstract] OR "lung cancer*"[Title/Abstract] OR "lung malignanc*"[Title/Abstract] OR "lung neoplas*"[Title/Abstract] OR "lung non small cell cancer"[Title/Abstract] OR "lung non small cell carcinoma"[Title/Abstract] OR "lung oat cell carcinoma"[Title/Abstract] OR "lung small cell cancer"[Title/Abstract] OR "lung small cell carcinoma"[Title/Abstract] OR "lung tumor*"[Title/Abstract] OR "lung tumour"[Title/Abstract] OR "malignancies of the lung"[Title/Abstract] OR "malignancy of the lung"[Title/Abstract] OR "malignant lung neoplasm"[Title/Abstract] OR "malignant lung tumor"[Title/Abstract] OR "malignant neoplasm of the lung"[Title/Abstract] OR "malignant tumor of the lung"[Title/Abstract] OR "microcellular lung carcinoma"[Title/Abstract] OR "neoplasia of the lung"[Title/Abstract] OR "neoplastic lung"[Title/Abstract] OR "non oat cell lung cancer"[Title/Abstract] OR "non small cell bronchial cancer"[Title/Abstract] OR "non small cell lung cancer"[Title/Abstract] OR "non small cell pulmonary cancer"[Title/Abstract] OR "non small cell pulmonary carcinoma"[Title/Abstract] OR "non squamous NSCLC"[Title/Abstract] OR "non-oat cell lung cancer"[Title/Abstract] OR "nonsmall cell carcinoma of the lung"[Title/Abstract] OR "nonsmall cell lung cancer"[Title/Abstract] OR "nonsmall cell lung carcinoma"[Title/Abstract] OR "non-small-cell lung carcinoma*"[Title/Abstract] OR "oat cell cancer of the lung"[Title/Abstract] OR "Oat Cell Carcinoma of Lung"[Title/Abstract] OR "oat cell carcinoma of the lung"[Title/Abstract] OR "oat cell lung cancer"[Title/Abstract] OR "oat cell lung carcinoma"[Title/Abstract] OR "pulmonary cancer*"[Title/Abstract] OR "pulmonary malignanc*"[Title/Abstract] OR "pulmonary neoplas*"[Title/Abstract] OR "pulmonary non small cell cancer"[Title/Abstract] OR "pulmonary non small cell carcinoma"[Title/Abstract] OR "pulmonary small cell cancer"[Title/Abstract] OR "pulmonary small cell carcinoma"[Title/Abstract] OR "pulmonary tumor*"[Title/Abstract] OR "pulmonary tumour"[Title/Abstract] OR "Schneeberg disease"[Title/Abstract] OR "Schneeberg lung disease"[Title/Abstract] OR "small cell bronchial cancer"[Title/Abstract] OR "small cell bronchial carcinoma"[Title/Abstract] OR "small cell cancer of the lung"[Title/Abstract] OR "small cell carcinoma of the lung"[Title/Abstract] OR "small cell lung cancer"[Title/Abstract] OR "small cell lung carcinoma"[Title/Abstract] OR "small cell lung tumor"[Title/Abstract] OR "small cell neuroendocrine carcinoma of the lung"[Title/Abstract] OR "small cell pulmonary cancer"[Title/Abstract] OR "small cell pulmonary carcinoma"[Title/Abstract] OR "tumor of the lung"[Title/Abstract] OR "tumorigenesis in the lung"[Title/Abstract] |
| 7 | (#1 OR #2) AND (#3 OR #4) AND (#5 OR #6) |
| 8 | "platelet-to-lymphocyte" OR "platelet/lymphocyte" OR "PLR" OR "monocyte-to-lymphocyte" OR "monocyte/lymphocyte" OR "MLR" OR "SII" OR "Systemic Immun* Inflamma*" OR "Neutrophi* to Lymphocy*" OR "Neutrophi*/Lymphocy*" OR "NLR" |
| 9 | #7 AND #8 |

| **Embase** | |
| --- | --- |
| # | Query |
| 1 | 'lung cancer'/exp OR 'lung cancer' OR 'lung tumor'/exp OR 'lung tumor' OR 'non small cell lung cancer'/exp OR 'non small cell lung cancer' OR 'small cell lung cancer'/exp OR 'small cell lung cancer' |
| 2 | 'bronchial non small cell cancer':ab,ti OR 'bronchial non small cell carcinoma':ab,ti OR 'bronchial small cell cancer':ab,ti OR 'bronchial small cell carcinoma':ab,ti OR 'bronchopulmonary cancer':ab,ti OR 'broncho-pulmonary cancer':ab,ti OR 'bronchopulmonary neoplas*':ab,ti OR 'broncho-pulmonary neoplasm':ab,ti OR 'bronchopulmonary tumor':ab,ti OR 'broncho-pulmonary tumor':ab,ti OR 'ca lung':ab,ti OR 'cancer of lung':ab,ti OR 'cancer of the lung':ab,ti OR 'carcinogenesis of the lung':ab,ti OR 'lung cancer*':ab,ti OR 'lung malignanc*':ab,ti OR 'lung neoplas*':ab,ti OR 'lung non small cell cancer':ab,ti OR 'lung non small cell carcinoma':ab,ti OR 'lung oat cell carcinoma':ab,ti OR 'lung small cell cancer':ab,ti OR 'lung small cell carcinoma':ab,ti OR 'lung tumor*':ab,ti OR 'lung tumour':ab,ti OR 'malignancies of the lung':ab,ti OR 'malignancy of the lung':ab,ti OR 'malignant lung neoplasm':ab,ti OR 'malignant lung tumor':ab,ti OR 'malignant neoplasm of the lung':ab,ti OR 'malignant tumor of the lung':ab,ti OR 'microcellular lung carcinoma':ab,ti OR 'neoplasia of the lung':ab,ti OR 'neoplastic lung':ab,ti OR 'non oat cell lung cancer':ab,ti OR 'non small cell bronchial cancer':ab,ti OR 'non small cell lung cancer':ab,ti OR 'non small cell pulmonary cancer':ab,ti OR 'non small cell pulmonary carcinoma':ab,ti OR 'non squamous nsclc':ab,ti OR 'non-oat cell lung cancer':ab,ti OR 'nonsmall cell carcinoma of the lung':ab,ti OR 'nonsmall cell lung cancer':ab,ti OR 'nonsmall cell lung carcinoma':ab,ti OR 'non-small-cell lung carcinoma*':ab,ti OR 'oat cell cancer of the lung':ab,ti OR 'oat cell carcinoma of lung':ab,ti OR 'oat cell carcinoma of the lung':ab,ti OR 'oat cell lung cancer':ab,ti OR 'oat cell lung carcinoma':ab,ti OR 'pulmonary cancer*':ab,ti OR 'pulmonary malignanc*':ab,ti OR 'pulmonary neoplas*':ab,ti OR 'pulmonary non small cell cancer':ab,ti OR 'pulmonary non small cell carcinoma':ab,ti OR 'pulmonary small cell cancer':ab,ti OR 'pulmonary small cell carcinoma':ab,ti OR 'pulmonary tumor*':ab,ti OR 'pulmonary tumour':ab,ti OR 'schneeberg disease':ab,ti OR 'schneeberg lung disease':ab,ti OR 'small cell bronchial cancer':ab,ti OR 'small cell bronchial carcinoma':ab,ti OR 'small cell cancer of the lung':ab,ti OR 'small cell carcinoma of the lung':ab,ti OR 'small cell lung cancer':ab,ti OR 'small cell lung carcinoma':ab,ti OR 'small cell lung tumor':ab,ti OR 'small cell neuroendocrine carcinoma of the lung':ab,ti OR 'small cell pulmonary cancer':ab,ti OR 'small cell pulmonary carcinoma':ab,ti OR 'tumor of the lung':ab,ti OR 'tumorigenesis in the lung':ab,ti |
| 3 | 'brain'/exp |
| 4 | 'brain*':ab,ti OR 'cerebr*':ab,ti OR 'encephalon':ab,ti |
| 5 | 'metastasis'/exp |
| 6 | 'cancer cell dissemination':ab,ti OR 'cancer cell spread':ab,ti OR 'cancer dissemination':ab,ti OR 'cancer spread':ab,ti OR 'disseminated tumor cell':ab,ti OR 'disseminated tumour cell':ab,ti OR 'metasta*':ab,ti OR 'neoplastic cell dissemination':ab,ti OR 'secondary cancer':ab,ti OR 'secondary carcinoma':ab,ti OR 'tumor dissemination':ab,ti OR 'tumor migration':ab,ti OR 'tumor spread':ab,ti OR 'tumour dissemination':ab,ti OR 'tumour migration':ab,ti OR 'tumour spread':ab,ti |
| 7 | 'platelet-to-lymphocyte' OR 'platelet/lymphocyte' OR 'plr' OR 'monocyte-to-lymphocyte' OR 'monocyte/lymphocyte' OR 'mlr' OR 'sii' OR 'systemic immun* inflamma*' OR 'neutrophi* to lymphocy*' OR 'neutrophi*/lymphocy*' OR 'nlr' |
| 8 | (#1 OR #2) AND (#3 OR #4) AND (#5 OR #6) AND #7 |

| **Cochrane** | |
| --- | --- |
| # | Query |
| 1 | MeSH descriptor: [Lung Neoplasms] explode all trees |
| 2 | (‘bronchial non small cell cancer’ OR ‘bronchial non small cell carcinoma’ OR ‘bronchial small cell cancer’ OR ‘bronchial small cell carcinoma’ OR ‘bronchopulmonary cancer’ OR ‘broncho-pulmonary cancer’ OR ‘bronchopulmonary neopla*’ OR ‘broncho-pulmonary neoplasm’ OR ‘bronchopulmonary tumor’ OR ‘broncho-pulmonary tumor’ OR ‘Ca lung’ OR ‘Cancer of Lung’ OR ‘cancer of the lung’ OR ‘carcinogenesis of the lung’ OR ‘Lung Cancer*’ OR ‘lung malignanc*’ OR ‘lung neopla*’ OR ‘lung non small cell cancer’ OR ‘lung non small cell carcinoma’ OR ‘lung oat cell carcinoma’ OR ‘lung small cell cancer’ OR ‘lung small cell carcinoma’ OR ‘lung tumor*’ OR ‘lung tumour’ OR ‘malignan* of the lung’ OR ‘malignant lung neoplasm’ OR ‘malignant lung tumor’ OR ‘malignant neoplasm of the lung’ OR ‘malignant tumor of the lung’ OR ‘microcellular lung carcinoma’ OR ‘neoplasia of the lung’ OR ‘neoplastic lung’ OR ‘non oat cell lung cancer’ OR ‘non small cell bronchial cancer’ OR ‘non small cell pulmonary cancer’ OR ‘non small cell pulmonary carcinoma’ OR ‘non squamous NSCLC’ OR ‘nonsmall cell carcinoma of the lung’ OR ‘nonsmall cell lung cancer’ OR ‘Non-Small Cell Lung Cancer*’ OR ‘nonsmall cell lung carcinoma’ OR ‘Non-Small Cell Lung Carcinoma*’ OR ‘oat cell cancer of the lung’ OR ‘Oat Cell Carcinoma of Lung’ OR ‘oat cell carcinoma of the lung’ OR ‘oat cell lung cancer’ OR ‘oat cell lung carcinoma’ OR ‘pulmonary cancer*’ OR ‘pulmonary malignan*’ OR ‘pulmonary neopla*’ OR ‘pulmonary non small cell cancer’ OR ‘pulmonary non small cell carcinoma’ OR ‘pulmonary small cell cancer’ OR ‘pulmonary small cell carcinoma’ OR ‘pulmonary tumor*’ OR ‘pulmonary tumour’ OR ‘Schneeberg disease’ OR ‘Schneeberg lung disease’ OR ‘small cell bronchial cancer’ OR ‘small cell bronchial carcinoma’ OR ‘small cell cancer of the lung’ OR ‘small cell carcinoma of the lung’ OR ‘small cell lung cancer’ OR ‘small cell lung carcinoma’ OR ‘small cell lung tumor’ OR ‘small cell neuroendocrine carcinoma of the lung’ OR ‘small cell pulmonary cancer’ OR ‘small cell pulmonary carcinoma’ OR ‘tumor of the lung’ OR ‘tumorigenesis in the lung’):ti,ab,kw |
| 3 | MeSH descriptor: [Brain] explode all trees |
| 4 | (‘Brain*’ OR ‘Cerebr*’ OR ‘encephalon’):ti,ab,kw |
| 5 | MeSH descriptor: [Neoplasm Metastasis] explode all trees |
| 6 | (‘cancer cell dissemination’ OR ‘cancer cell spread’ OR ‘cancer dissemination’ OR ‘cancer spread’ OR ‘disseminated tumor cell’ OR ‘disseminated tumour cell’ OR ‘Metasta*’ OR ‘neoplastic cell dissemination’ OR ‘secondary cancer’ OR ‘secondary carcinoma’ OR ‘tumor dissemination’ OR ‘tumor migration’ OR ‘tumor spread’ OR ‘tumour dissemination’ OR ‘tumour migration’ OR ‘tumour spread’):ti,ab,kw |
| 7 | (‘platelet to lymphocyte’ OR ‘PLR’ OR ‘monocyte to lymphocyte’ OR ‘MLR’ OR ‘SII’ OR ‘Systemic Immun* Inflamma*’ OR ‘Neutrophi* to Lymphocy*’ OR ‘NLR’) |
| 8 | (#1 OR #2) AND (#3 OR #4) AND (#5 OR #6) AND #7 |

| **Web of Science** | |
| --- | --- |
| # | Query |
| 1 | TS=((brain*) OR (Cerebr*) OR (encephalon)) and Preprint Citation Index (Exclude – Database) and Research Commons (Exclude – Database) |
| 2 | TS=((cancer cell dissemination) OR (cancer cell spread) OR (cancer dissemination) OR (cancer spread) OR (disseminated tumor cell) OR (disseminated tumour cell) OR (Metasta*) OR (secondary cancer) OR (secondary carcinoma) OR (tumor dissemination) OR (tumor migration) OR (tumor spread) OR (tumour dissemination) OR (tumour migration) OR (tumour spread)) and Preprint Citation Index (Exclude – Database) and Research Commons (Exclude – Database) |
| 3 | TS=((bronchial non small cell cancer) OR (bronchial non small cell carcinoma) OR (bronchial small cell cancer) OR (bronchial small cell carcinoma) OR (bronchopulmonary cancer) OR (broncho-pulmonary cancer) OR (bronchopulmonary neopla*) OR (broncho-pulmonary neoplasm) OR (bronchopulmonary tumor) OR (broncho-pulmonary tumor) OR (Ca lung) OR (Cancer of Lung) OR (cancer of the lung) OR (carcinogenesis of the lung) OR (Lung Cancer*) OR (lung malignanc*) OR (lung neopla*) OR (lung non small cell cancer) OR (lung non small cell carcinoma) OR (lung oat cell carcinoma) OR (lung small cell cancer) OR (lung small cell carcinoma) OR (lung tumor*) OR (lung tumour) OR (malignan* of the lung) OR (malignant lung neoplasm) OR (malignant lung tumor) OR (malignant neoplasm of the lung) OR (malignant tumor of the lung) OR (microcellular lung carcinoma) OR (neoplasia of the lung) OR (neoplastic lung) OR (non oat cell lung cancer) OR (non small cell bronchial cancer) OR (non small cell pulmonary cancer) OR (non small cell pulmonary carcinoma) OR (non squamous NSCLC) OR (nonsmall cell carcinoma of the lung) OR (nonsmall cell lung cancer) OR (Non-Small Cell Lung Cancer*) OR (nonsmall cell lung carcinoma) OR (Non-Small Cell Lung Carcinoma*) OR (oat cell cancer of the lung) OR (Oat Cell Carcinoma of Lung) OR (oat cell carcinoma of the lung) OR (oat cell lung cancer) OR (oat cell lung carcinoma) OR (pulmonary cancer*) OR (pulmonary malignan*) OR (pulmonary neopla*) OR (pulmonary non small cell cancer) OR (pulmonary non small cell carcinoma) OR (pulmonary small cell cancer) OR (pulmonary small cell carcinoma) OR (pulmonary tumor*) OR (pulmonary tumour) OR (Schneeberg disease) OR (Schneeberg lung disease) OR (small cell bronchial cancer) OR (small cell bronchial carcinoma) OR (small cell cancer of the lung) OR (small cell carcinoma of the lung) OR (small cell lung cancer) OR (small cell lung carcinoma) OR (small cell lung tumor) OR (small cell neuroendocrine carcinoma of the lung) OR (small cell pulmonary cancer) OR (small cell pulmonary carcinoma) OR (tumor of the lung) OR (tumorigenesis in the lung)) and Preprint Citation Index (Exclude – Database) and Research Commons (Exclude – Database) |
| 4 | TS=((platelet-to-lymphocyte) OR (platelet/lymphocyte) OR (PLR) OR (monocyte-to-lymphocyte) OR (monocyte/lymphocyte) OR (MLR) OR (SII) OR (Systemic Immun* Inflamma*) OR (Neutrophi* to Lymphocy*) OR (Neutrophi*/Lymphocy*) OR (NLR)) and Preprint Citation Index (Exclude – Database) and Research Commons (Exclude – Database) |
| 5 | #1 AND #2 AND #3 AND #4 and Preprint Citation Index (Exclude – Database) and Research Commons (Exclude – Database) |

| **Scopus** | |
| --- | --- |
| # | Query |
| 1 | TITLE-ABS-KEY ( "brain*" OR "Cerebr*" OR "encephalon" ) |
| 2 | TITLE-ABS-KEY ( "cancer cell dissemination" OR "cancer cell spread" OR "cancer dissemination" OR "cancer spread" OR "disseminated tumor cell" OR "disseminated tumour cell" OR "Metasta*" OR "secondary cancer" OR "secondary carcinoma" OR "tumor dissemination" OR "tumor migration" OR "tumor spread" OR "tumour dissemination" OR "tumour migration" OR "tumour spread" ) |
| 3 | TITLE-ABS-KEY ( "lung cancer*" OR "lung tumor*" OR "lung neopla*" ) |
| 4 | TITLE-ABS-KEY ( "platelet-to-lymphocyte" OR "platelet/lymphocyte" OR "PLR" OR "monocyte-to-lymphocyte" OR "monocyte/lymphocyte" OR "MLR" OR "SII" OR "Systemic Immun* Inflamma*" OR "Neutrophi* to Lymphocy*" OR "Neutrophi*/Lymphocy*" OR "NLR" ) ) |
| 5 | #1 AND #2 AND #3 AND #4 |

**Table S2.** Newcastle-Ottawa Scale (NOS)

| **Study** | **Selection** | | | | **Comparability** | **Outcome** | | | **Overall Score** |
| --- | --- | --- | --- | --- | --- | --- | --- | --- | --- |
|  | **Representativeness of the Exposed Cohort** | **Selection of the Non-Exposed Cohort** | **Ascertainment of Exposure** | **Demonstration That Outcome of Interest Was Not Present at Start of Study** | **Comparability of Cohorts on the Basis of the Design or Analysis** | **Assessment of Outcome** | **Was Follow-Up Long Enough for Outcomes to Occur?** | **Adequacy of Follow-Up of Cohorts** |  |
| Zhou2025 | 1 | - | 1 | 1 | 2 | 1 | 1 | 1 | 8 |
| Marschollek2025 | 1 | - | 1 | 1 | 1 | 1 | 1 | 1 | 7 |
| Li2023 | 1 | - | 1 | - | 1 | 1 | 1 | 1 | 6 |
| Abuelbeh2022 | 1 | - | 1 | - | 2 | 1 | 1 | 1 | 7 |
| Liu2022 | 1 | - | 1 | - | 2 | 1 | 1 | 1 | 7 |
| Wang2017 | 1 | - | 1 | 1 | 2 | 1 | 1 | 1 | 8 |
| Koh2016 | 1 | - | 1 | - | 2 | 1 | 1 | 1 | 7 |
| Sert2021 | 1 | - | 1 | 1 | 2 | 1 | 1 | 1 | 8 |
| Chung2020 | 1 | - | 1 | 1 | 2 | 1 | 1 | 1 | 8 |
| Gu2024 | 1 | - | 1 | - | 1 | 1 | 1 | 1 | 6 |
| Hou2022 | 1 | - | 1 | 1 | 2 | 1 | 1 | 1 | 8 |
| Hu2022 | 1 | - | 1 | - | 2 | 1 | 1 | 1 | 7 |
| Qiu2022 | 1 | - | 1 | 1 | 2 | 1 | 1 | 1 | 8 |
| Suzuki2018 | 1 | - | 1 | 1 | 2 | 1 | 1 | 1 | 8 |


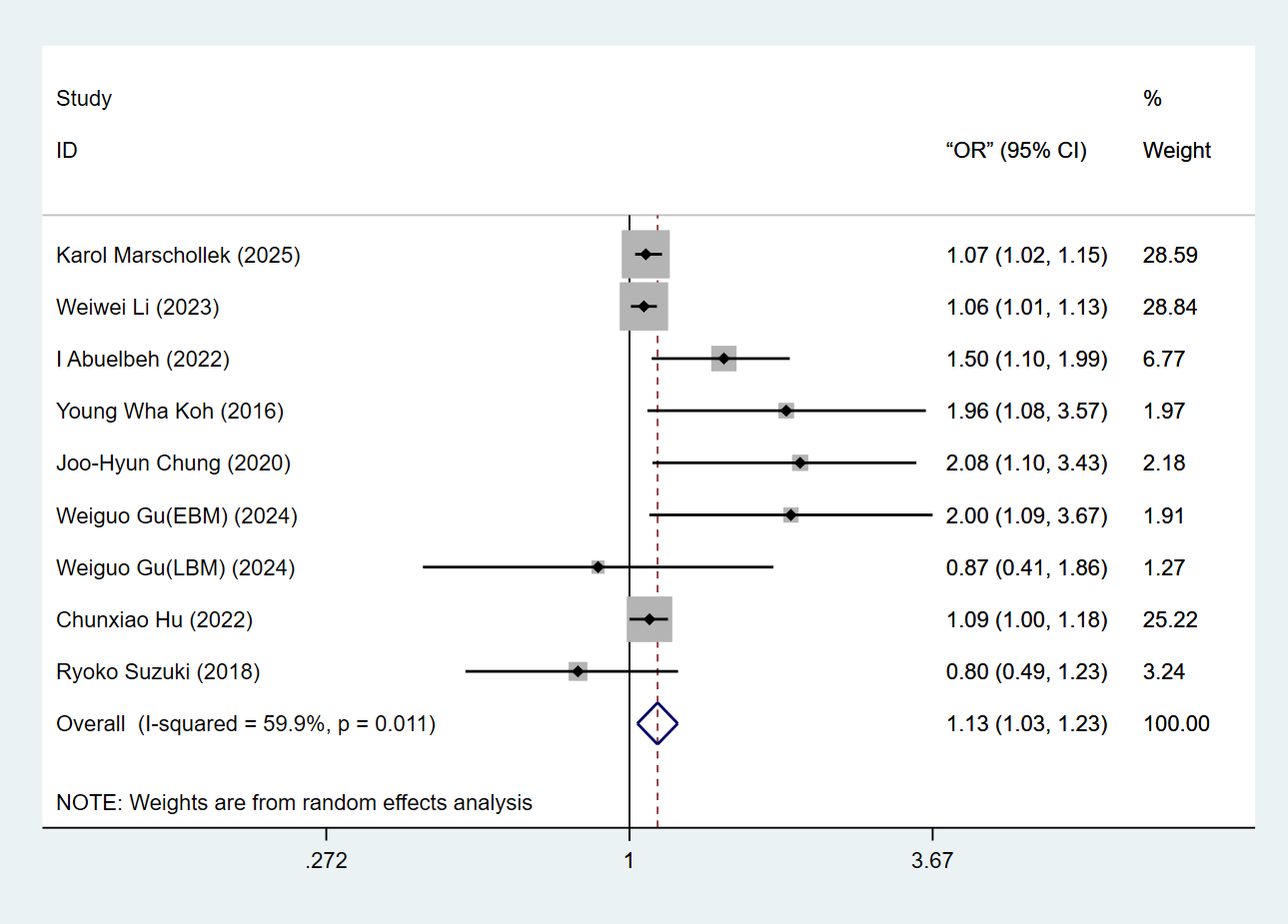


**Figure S1.** Forest plot of univariate analyses on the association between high neutrophil-to-lymphocyte ratio and brain metastasis from lung cancer


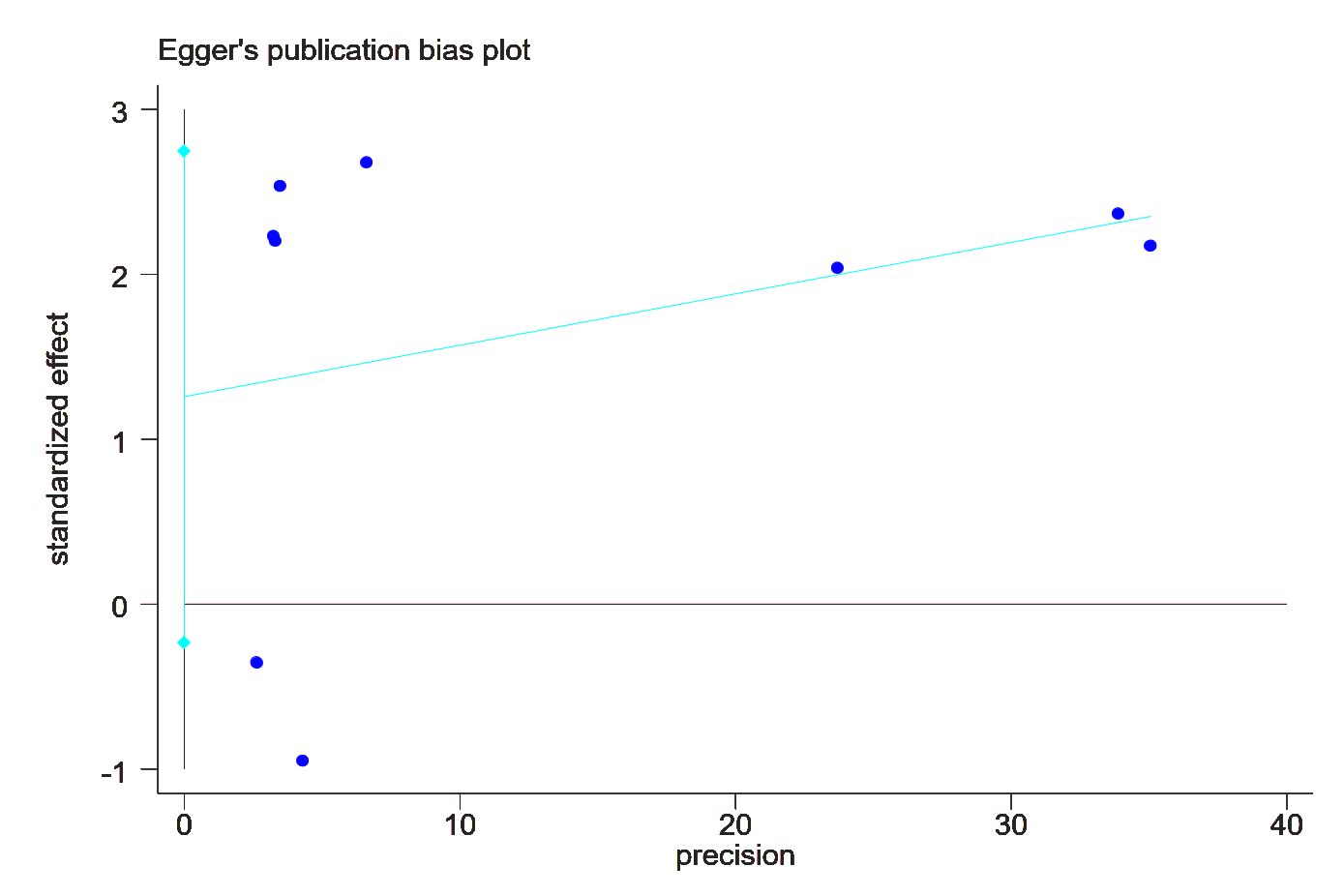


**Figure S2.** Egger's publication bias plot for univariate analyses on the association between high neutrophil-to-lymphocyte ratio and brain metastasis from lung cancer


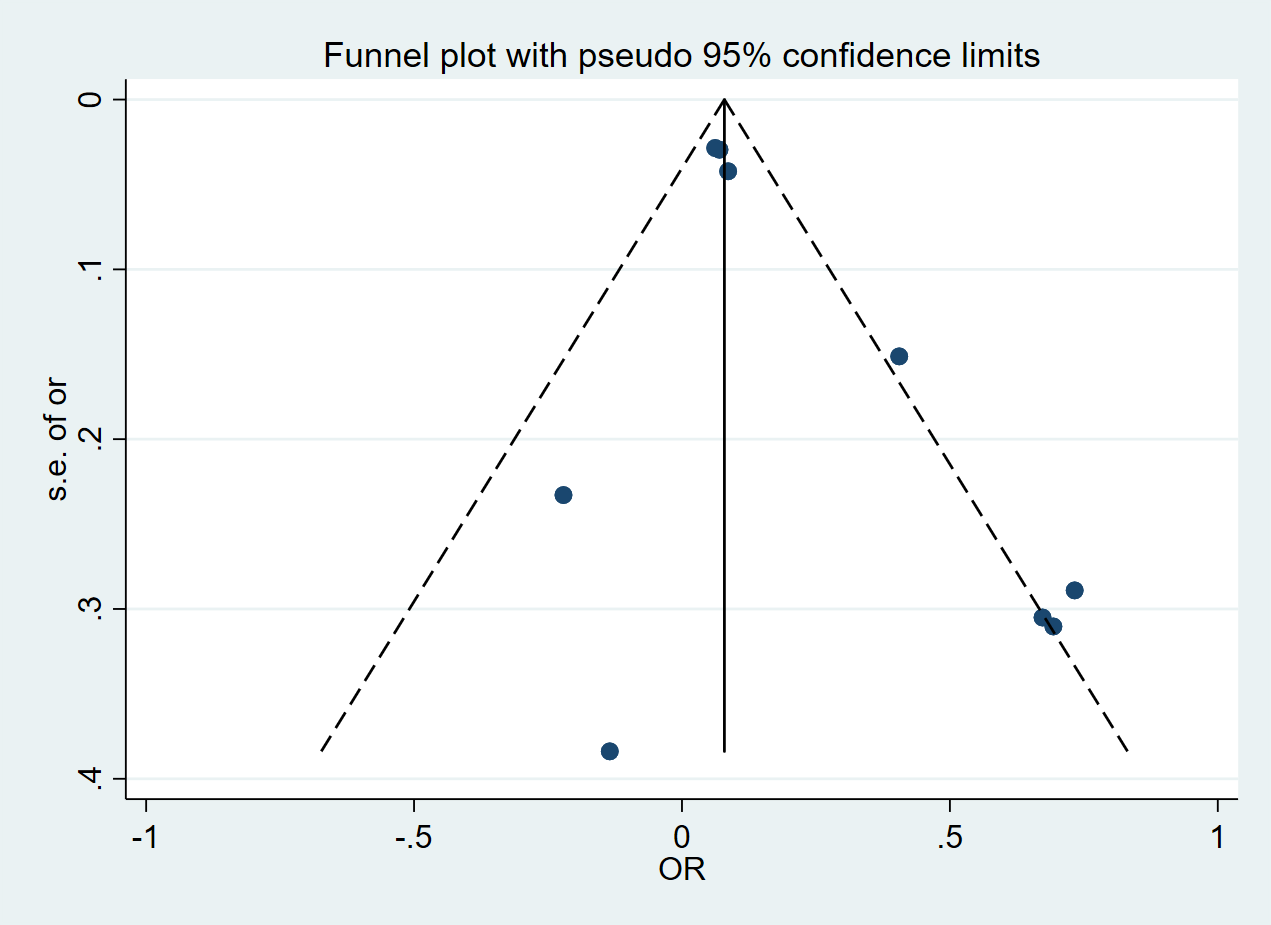


**Figure S3.** Funnel plot of publication bias for univariate analyses on the association between high neutrophil-to-lymphocyte ratio and brain metastasis from lung cancer


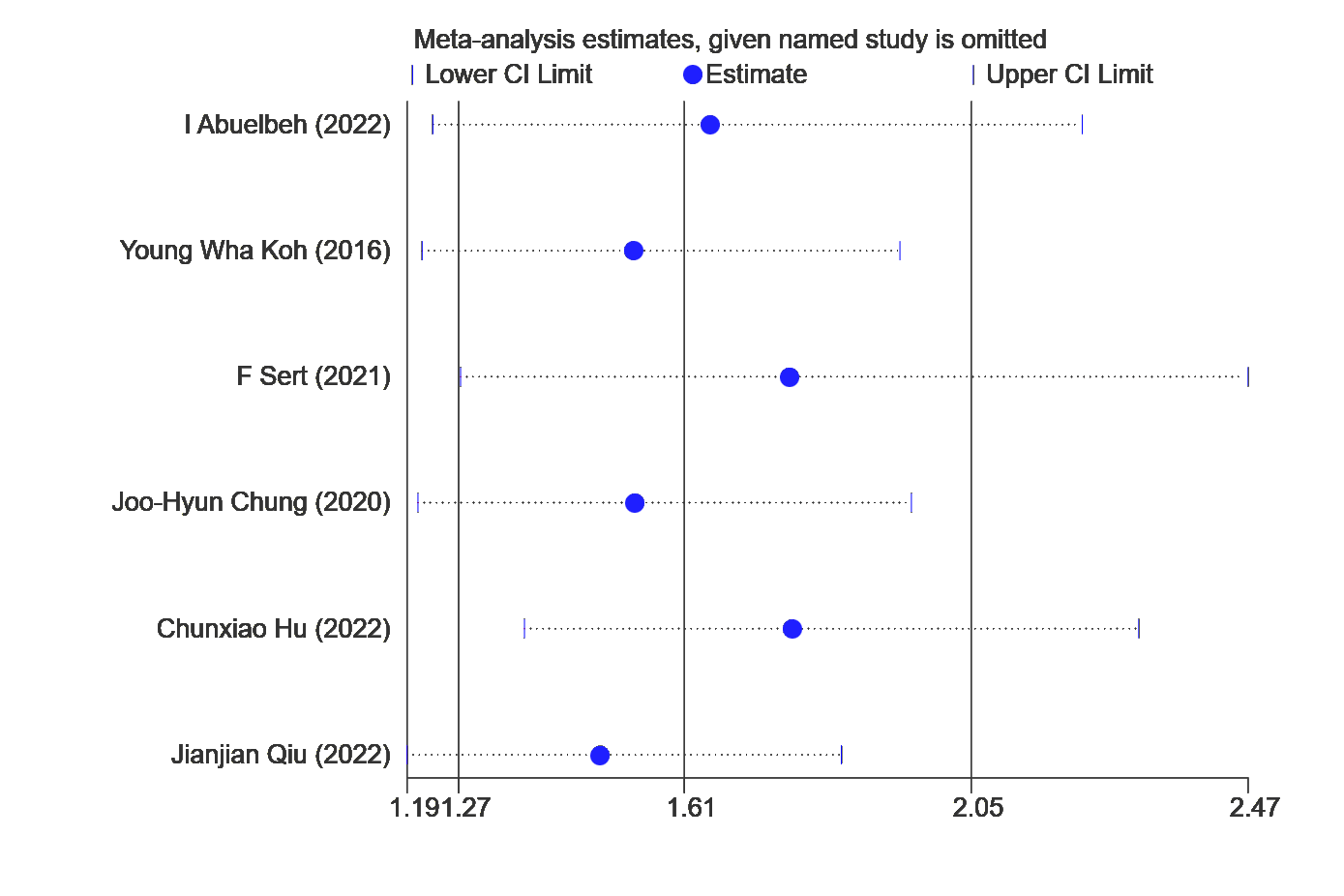


**Figure S4.** Forest plot of sensitivity analysis for results from multivariate analyses on the association between high neutrophil-to-lymphocyte ratio and brain metastasis from lung cancer (one-by-one exclusion method)


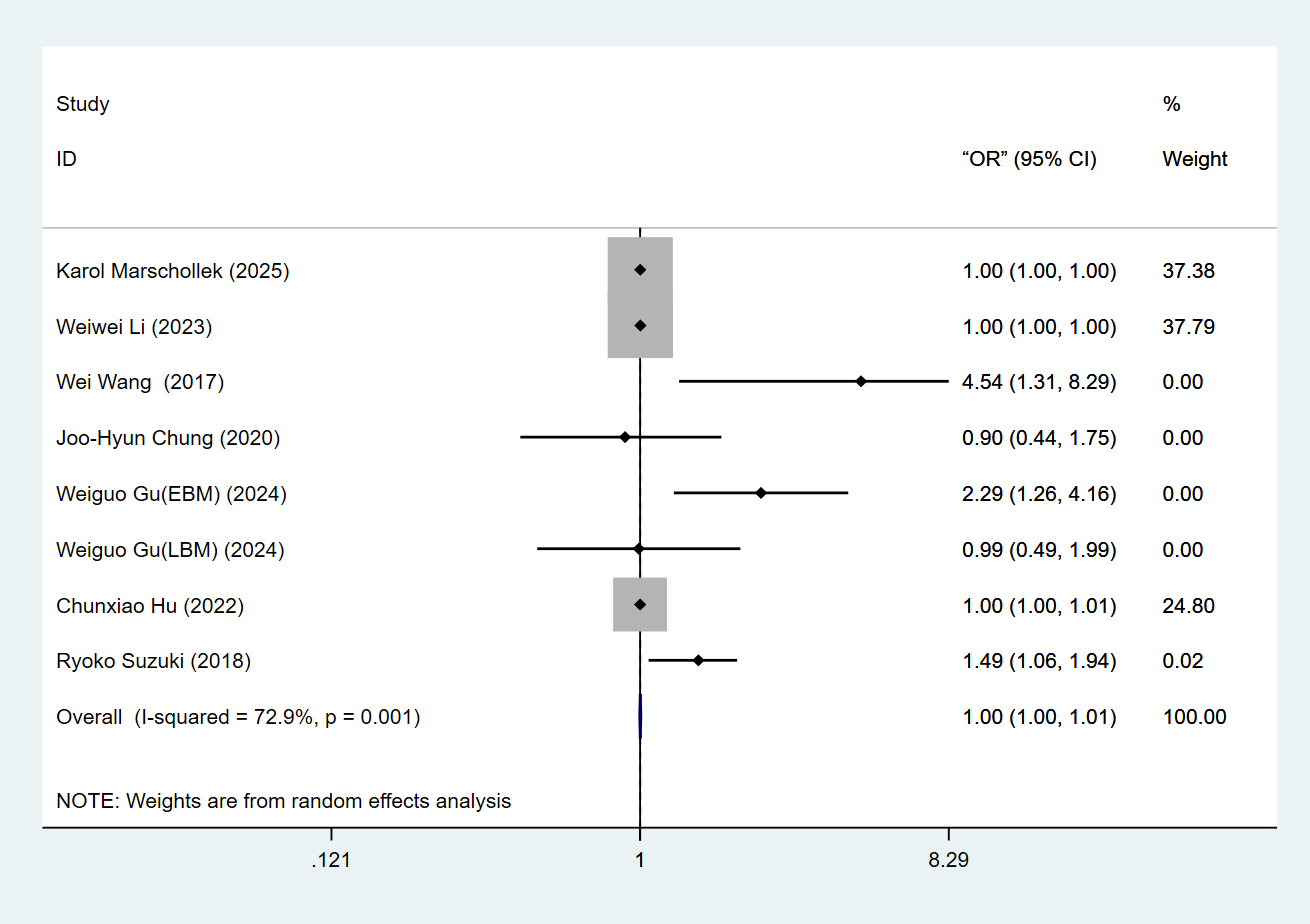


**Figure S5.** Forest plot of univariate analysis for the association between high platelet-to-lymphocyte ratio and brain metastasis from lung cancer


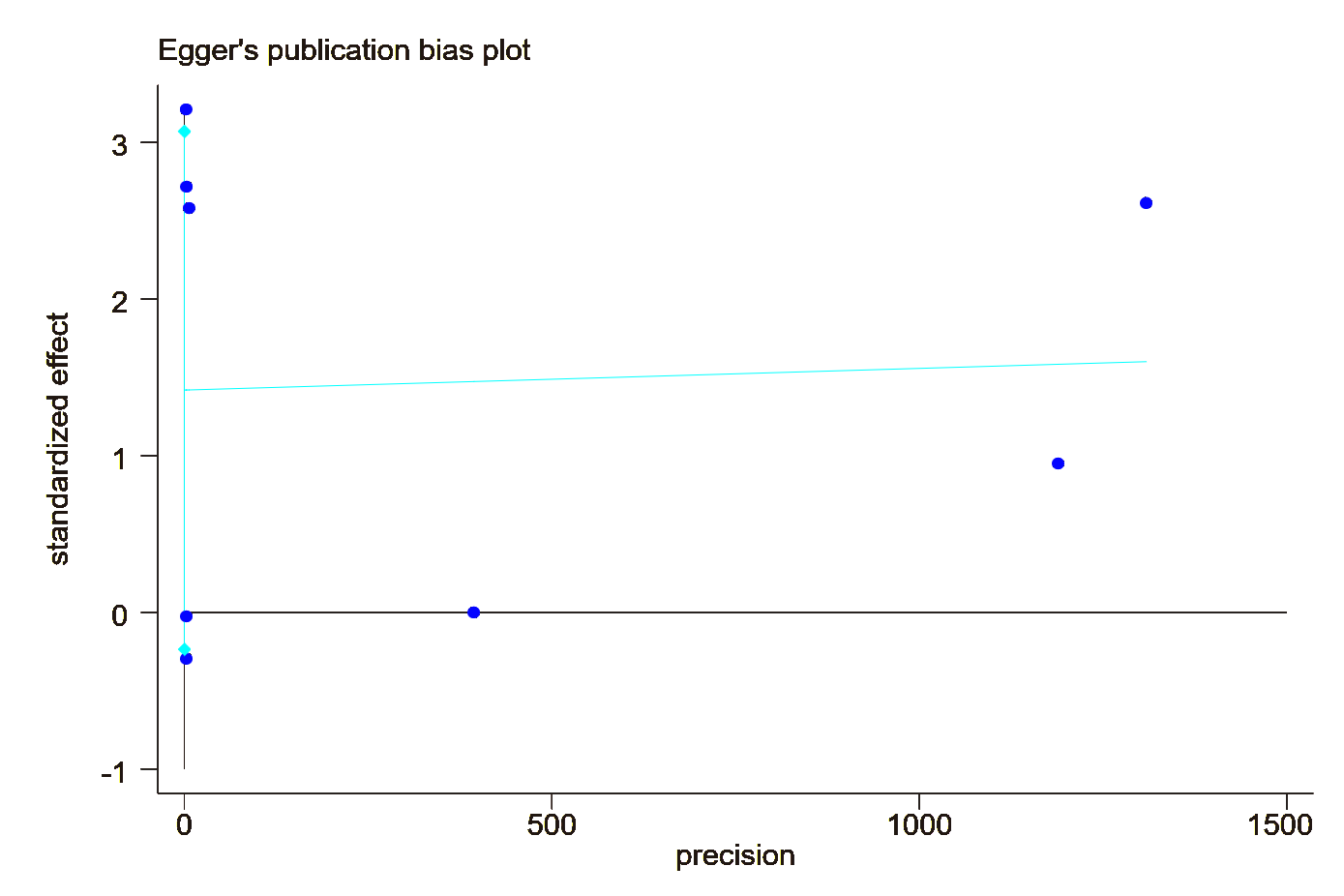


**Figure S6.** Egger's publication bias plot for univariate analyses on the association between high platelet-to-lymphocyte ratio and brain metastasis from lung cancer


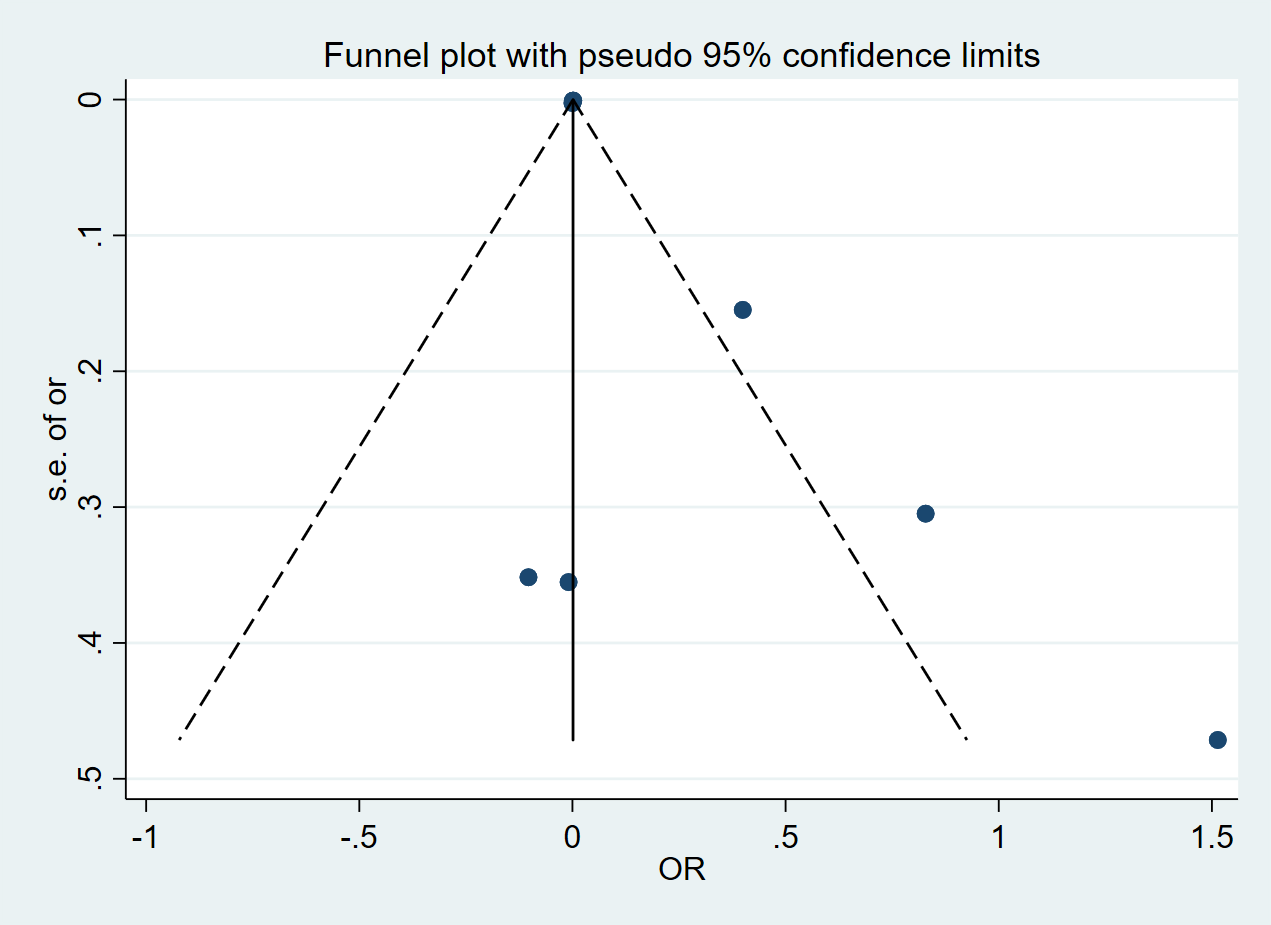


**Figure S7.** Funnel plot of publication bias for univariate analyses on the association between high platelet-to-lymphocyte ratio and brain metastasis from lung cancer


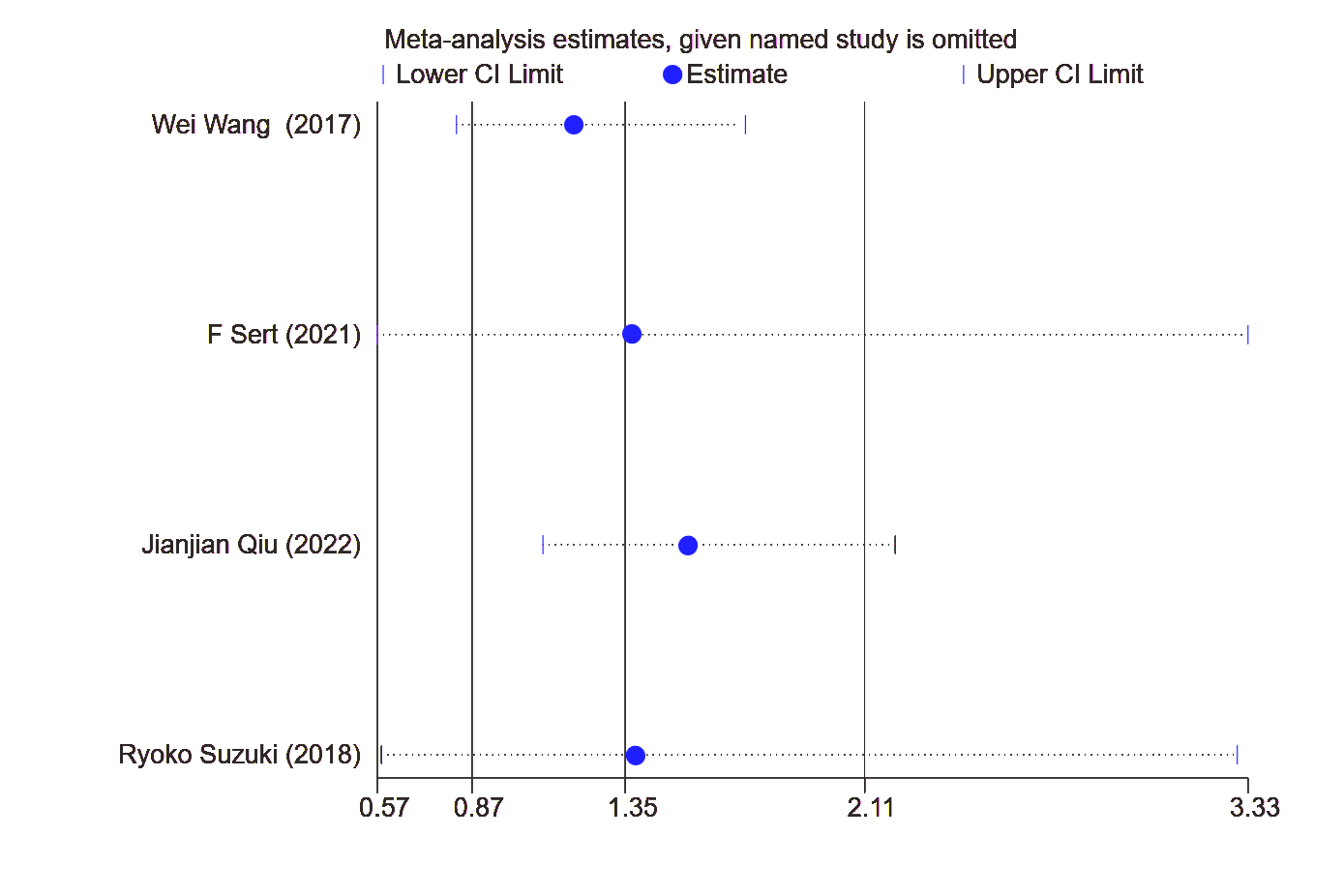


**Figure S8.** Forest plot of sensitivity analysis for results from multivariate analyses on the association between high platelet-to-lymphocyte ratio and brain metastasis from lung cancer (one-by-one exclusion method)


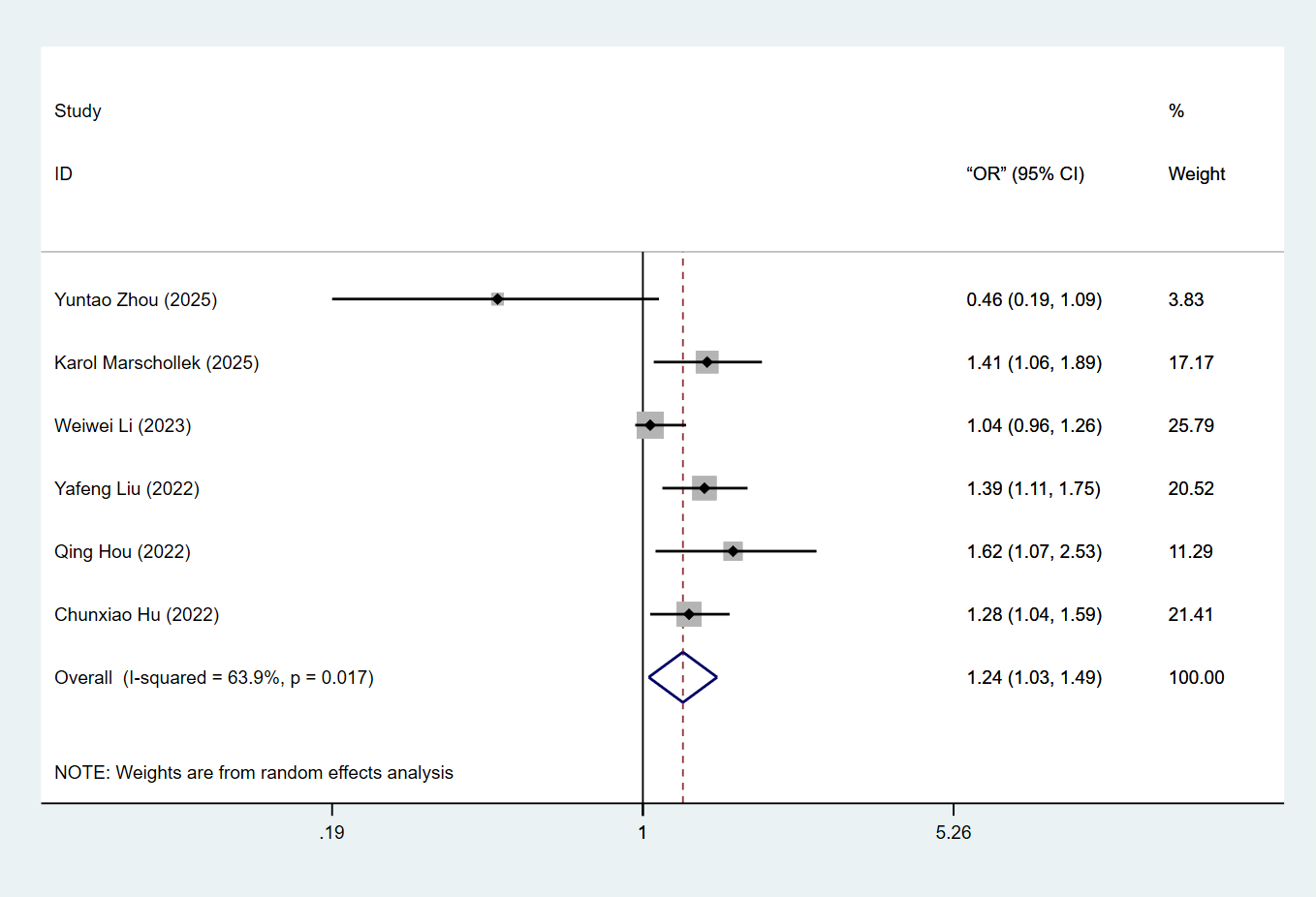


**Figure S9.** Forest plot of univariate analysis for the association between low lymphocyte-to-monocyte ratio and brain metastasis from lung cancer


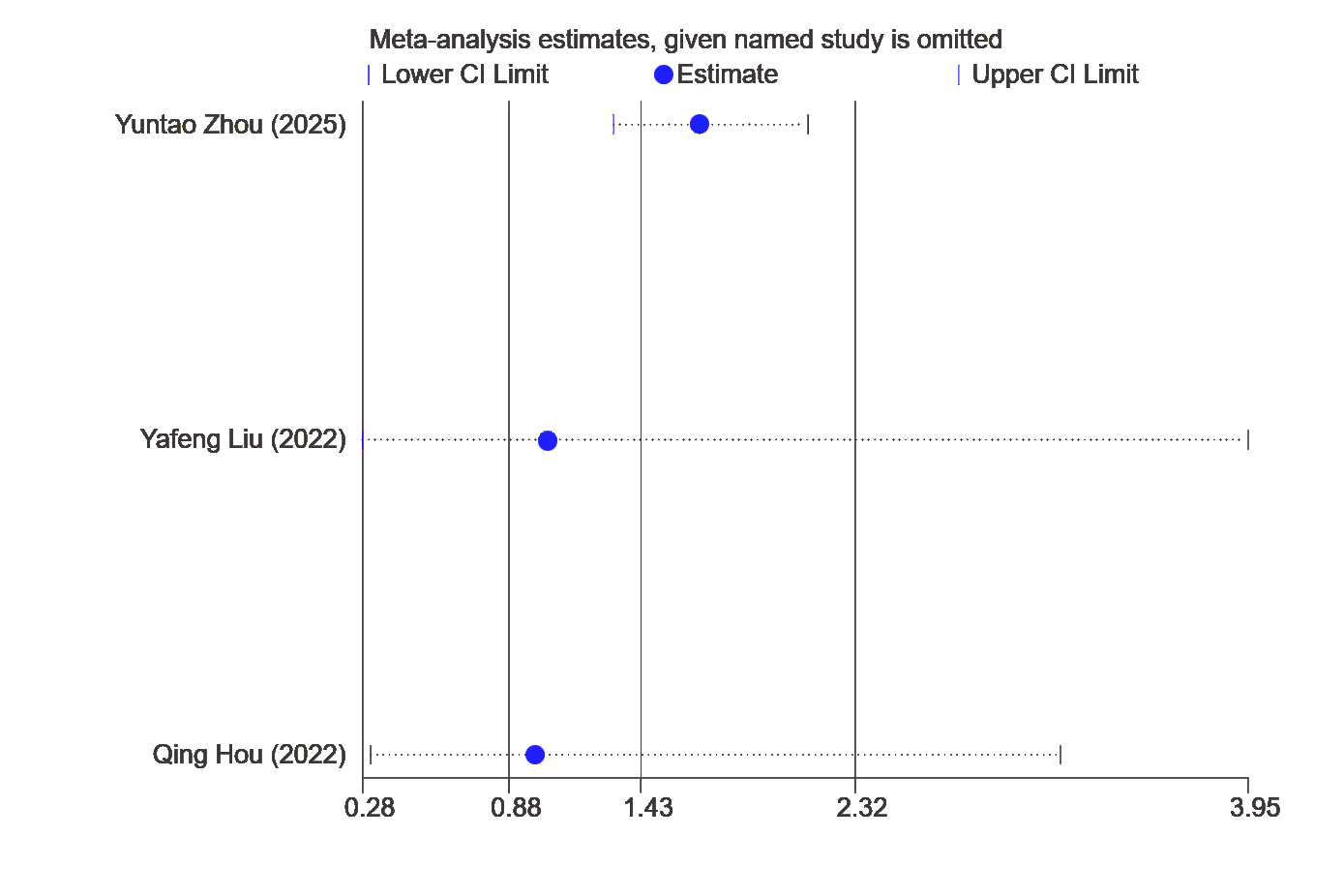


**Figure S10.** Forest plot of sensitivity analysis for results from multivariate analyses on the association between low lymphocyte-to-monocyte ratio and brain metastasis from lung cancer (one-by-one exclusion method)

Additional Tables

| **Author** | **Year** | **Cut-off** | | |
| --- | --- | --- | --- | --- |
|  |  | **NLR** | **PLR** | **LMR** |
| Yuntao Zhou[21] | 2025 | N/A | N/A | 3.90* |
| Karol Marschollek[22] | 2025 | 4.56* | N/R | 1.66* |
| Weiwei Li[23] | 2023 | 2.91 [1.70-4.65]*** | 183.43 [111.18-257.14]*** | 2.92 [1.74-4.42]*** |
| I Abuelbeh[24] | 2022 | 4.3* | N/A | N/A |
| Yafeng Liu[25] | 2022 | N/A | N/A | N/R |
| Wei Wang [26] | 2017 | N/A | 120* | N/A |
| Young Wha Koh[27] | 2016 | 4.95* | N/A | N/A |
| F Sert[28] | 2021 | 2.6* | 198* | N/A |
| Joo-Hyun Chung[29] | 2020 | 3.7* | 151.2* | N/A |
| Weiguo Gu[30] | 2024 | 3.68***** | 184***** | N/A |
| Qing Hou[31] | 2022 | N/A | N/A | 2.70**** |
| Chunxiao Hu[32] | 2022 | 5.52±4.24** | 213.31±97.74** | 2.61±1.13** |
| Jianjian Qiu[33] | 2022 | 3.23**** | 97.3**** | N/A |
| Ryoko Suzuki[34] | 2018 | 1.6* | 119.4* | N/A |

*:Based on ROC curve analysis，**:Mean ± standard deviation (SD),***: Median (interquartile range, IQR),****: Maximally selected rank statistics,*****:Mean value, N/A: Not Available, N/R: Not Reported
